# A PSTAIRE-type cyclin-dependent kinase controls light responses in land plants

**DOI:** 10.1126/sciadv.abk2116

**Published:** 2022-01-28

**Authors:** Liang Bao, Natsumi Inoue, Masaki Ishikawa, Eiji Gotoh, Ooi-Kock Teh, Takeshi Higa, Tomoro Morimoto, Eggie Febrianto Ginanjar, Hirofumi Harashima, Natsumi Noda, Masaaki Watahiki, Yuji Hiwatashi, Masami Sekine, Mitsuyasu Hasebe, Masamitsu Wada, Tomomichi Fujita

**Affiliations:** 1 Graduate School of Life Science, Hokkaido University, Sapporo 060-0810, Japan.; 2 Division of Evolutionary Biology, National Institute for Basic Biology, Okazaki 444-8585, Japan.; 3 School of Life Science, SOKENDAI (The Graduate University for Advanced Studies), Okazaki 444-8585, Japan.; 4 Faculty of Agriculture, Kyushu University, Fukuoka 819-0395, Japan.; 5 Institute for the Advancement of Higher Education, Hokkaido University, Sapporo 060-0817, Japan.; 6 Faculty of Science, Kyushu University, Fukuoka 812-8581, Japan.; 7 Cell Function Research Team, RIKEN Centre for Sustainable Resource Science, Yokohama 230-0045, Japan.; 8 Faculty of Science, Hokkaido University, Sapporo 060-0810, Japan.; 9 School of Food Industrial Sciences, Miyagi University, Sendai 982-0215, Japan.; 10 Faculty of Bioresources and Environmental Sciences, Ishikawa Prefectural University, Nonoichi 921-8836, Japan.

## Abstract

Light is a critical signal perceived by plants to adapt their growth rate and direction. Although many signaling components have been studied, how plants respond to constantly fluctuating light remains underexplored. Here, we showed that in the moss *Physcomitrium* (*Physcomitrella*) *patens*, the PSTAIRE-type cyclin-dependent kinase PpCDKA is dispensable for growth. Instead, PpCDKA and its homolog in *Arabidopsis thaliana* control light-induced tropisms and chloroplast movements by probably influencing the cytoskeleton organization independently of the cell cycle. In addition, lower PpCDKA kinase activity was required to elicit light responses relative to cell cycle regulation. Thus, our study suggests that plant CDKAs may have been co-opted to control multiple light responses, and owing to the bistable switch properties of PSTAIRE-type CDKs, the noncanonical functions are widely conserved for eukaryotic environmental adaptation.

## Teaser

CDKA controls light-induced tropisms and chloroplast movement independently of cell cycle function in plants.

## INTRODUCTION

Land plants and other photosynthetic organisms produce sugars from harvesting sunlight, which changes daily and seasonally in quality, quantity, direction, and duration. Thus, light is one of the most variable environmental factors influencing plant growth (*1*). Accordingly, land plants have evolved sophisticated light-sensing mechanisms to optimize light harvesting in these ever-changing light conditions (*1*–*3*). Although the complement of photoreceptors and multiple downstream signaling components have been uncovered and their roles described under constant light conditions, the molecular mechanisms underlying responses to changing light conditions remain to be elucidated.

The core cell cycle regulator PSTAIRE-type cyclin-dependent kinase A (CDKA) in plants and its homologs in yeast (Cdc2 in *Schizosaccharomyces pombe* and CDC28 in *Saccharomyces cerevisiae*) and mammals (CDK1) play indispensable roles in cell cycle progression and development. Their inactivation causes cell cycle arrest or severe defects during embryogenesis (*4*–*6*). In the past two decades, non–cell cycle functions of nonessential and non–PSTAIRE-type CDKs have also been described in animal postmitotic cells during interphase (*7*–*9*). Likewise, functions for PSTAIRE-type CDKs outside the cell cycle have been reported; however, the cell mortality and severe developmental defects associated with loss of CDK function have prevented a conclusive dissection of their non–cell cycle roles (*10*–*13*). Here, we provide unambiguous evidence for non–cell cycle functions of PSTAIRE-type CDKAs in the moss *Physcomitrium* (*Physcomitrella*) *patens*. Whereas *P. patens* CDKA null mutants are viable, the mutants are deficient for multiple light responses that are independent of cell cycle progression. Thus, our results further accelerate the study for noncanonical functions of PSTAIRE-type CDKs on environmental responses in many eukaryotes.

## RESULTS

### PpCDKAs are dispensable for growth and morphogenesis

**Fig. 1.**
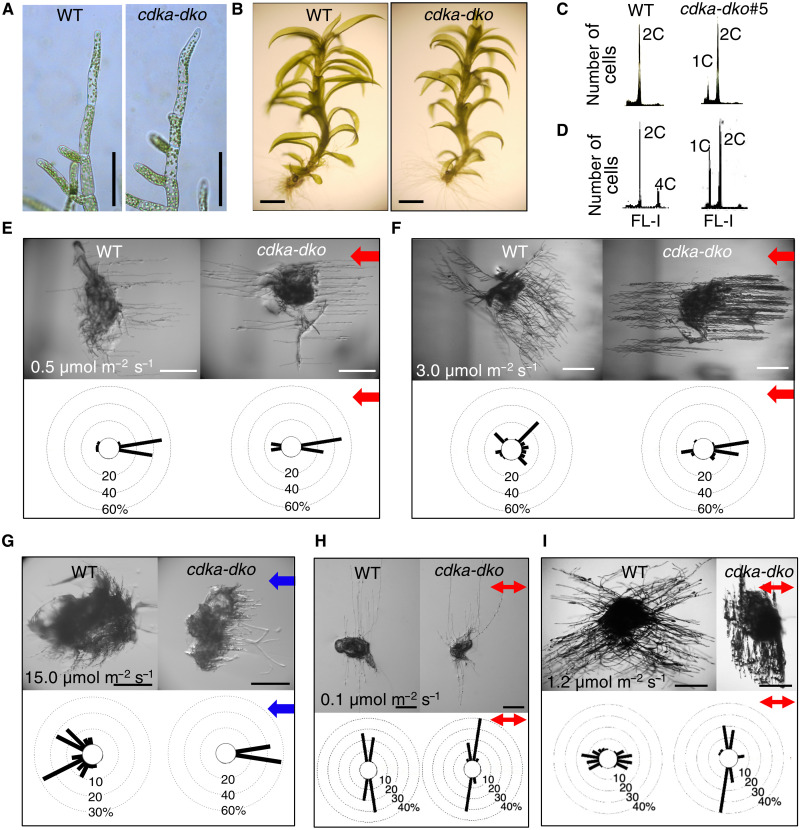
PpCDKAs control cell cycle progression and phototropic and polarotropic responses in the moss *P. patens*. (**A** and **B**) Representative images of wild-type (WT; left) and *cdka-dko* (right) protonemata (A) and gametophores (B). (**C** and **D**) Ploidy profiles of protonemata (C) and gametophores (D). (**E** to **I**) Representative images of light-induced tropism. Graphs (bottom) show the percentage distribution of orientations adopted by protonemal filaments. (E and F) PpCDKAs control unilateral red light (RL)–induced tropism. Protonemata distributions are based on 37 (WT) and 42 (*cdka-dko*) protonemal filaments exposed to 0.5 μmol m^−2^ s^−1^ RL (E), or 74 (WT) and 58 (*cdka-dko*) exposed to 3.0 μmol m^−2^ s^−1^ RL (F). (G) PpCDKAs control blue light (BL)–induced tropism of protonemata. The WT (*n* = 43; left) and *cdka-dko* (*n* = 46; right) protonemata were illuminated with unilateral BL at 15.0 μmol m^−2^ s^−1^. (H and I) Polarized RL-induced tropism by PpCDKAs (double-headed arrows indicate the E-vector). Protonemata distributions are based on 46 (WT) and 36 (*cdka-dko*) protonemal filaments illuminated with 0.1 μmol m^−2^ s^−1^ polarized RL (H), or 84 (WT) and 54 (*cdka-dko*) protonemal filaments illuminated with 1.2 μmol m^−2^ s^−1^ polarized RL (I). Scale bars, 100 μm (A) and 1 mm (B to I).

The moss *P. patens* offers many technical advantages over typical land plant models, including a simple body plan and highly efficient homologous recombination (*14*, *15*). The *P. patens* genome encodes two PSTAIRE-type CDKAs: PpCDKA;1 and PpCDKA;2 (*16*). To understand their functions, we generated a *CDKA* double knockout mutant (*cdka-dko*) by homologous recombination (fig. S1, A to C). Unexpectedly, *cdka-dko* protonemata and gametophores developed almost normally, resembling those from the wild type (WT) (Fig. 1, A and B), in sharp contrast to PSTAIRE-type *cdk* mutants reported in all other multicellular eukaryotes (*4*, *6*, *17*). To determine whether PpCDKA;1 and PpCDKA;2 are functional PSTAIRE-type CDKs, we attempted to complement the yeast temperature-sensitive *cdc28-4* mutant (*18*) by heterologous expression of *PpCDKA;1* and *PpCDKA;*2. We also examined two of the seven *PpCDKB* genes crucial for cell cycle progression: the plant-specific, non–PSTAIRE-type CDKs PpCDKB;1 and PpCDKB;2 (*19*). Expression of either *PpCDKA* rescued the cell cycle defects in *cdc28-4* mutant, whereas expression of the *PpCDKB*s did not (fig. S1D), demonstrating that PpCDKAs, but not PpCDKBs, are functional PSTAIRE-type CDKs. Next, we performed flow cytometry on nuclei from the WT and the *cdka-dko* mutant, which revealed a delay at the G1/S transition in mutant protonemal tissues and gametophores, with a distinct peak representing 1C cells (Fig. 1, C and D). Thus, PpCDKAs control cell cycle progression in moss, as in other plants (*6*, *19*). Yet, loss of PpCDKA function is survivable, as in the unicellular green alga *Chlamydomonas reinhardtii* (*20*), but unlike in yeasts, mammals, and other land plants.

### PpCDKAs mediate light responses

We noticed that WT protonemata showed negative phototropic growth by growing away from strong illumination (white light, ~50 μmol m^−2^ s^−1^), whereas some protonemata from the *cdka-dko* mutant showed positive phototropism (fig. S2A). To test whether the observed growth defect is a light-specific response, we incubated WT and *cdka-dko* plants in the dark; under this condition, both genotypes showed negative gravitropism (fig. S2B). This result suggested that the directional growth defect in *cdka-dko* mutants involves a light-specific response, prompting us to further explore the roles of CDKAs in light responses.

Moss protonemata exhibit light-induced tropisms, such as phototropism (directional growth in response to unilateral light) and polarotropism [directional growth relative to the electric field vector (E-vector) of polarized light], when grown in red light (RL) or blue light (BL) (*21*). WT protonemata grew toward weak RL but showed a consistent ~30° to 80° growth angle relative to the light direction under stronger RL (Fig. 1, E and F, and fig. S3A). They showed little phototropism under weak unilateral BL and negative phototropism under strong unilateral BL (Fig. 1G and fig. S3B). By contrast, the growth of *cdka-dko* protonemata was parallel to incident light at all intensities of RL and BL tested (Fig. 1, E to G, and fig. S3, A and B). We repeated these experiments with polarized RL or BL. Under weak polarized RL, WT protonemata grew perpendicular to the E-vector of polarized light but reoriented to become more parallel to the E-vector at higher light intensities (Fig. 1, H and I, and fig. S3C). Similarly, under polarized BL, WT protonemata adjusted the direction of their apical cells according to light intensity (fig. S3D). However, *cdka-dko* plants did not respond to intensity changes of polarized light, as protonemata grew perpendicular to the E-vector of polarized light at all RL and BL intensities tested (Fig. 1, H and I, and fig. S3, C and D). We concluded that PpCDKAs contribute to both phototropism and polarotropism.

**Fig. 2.**
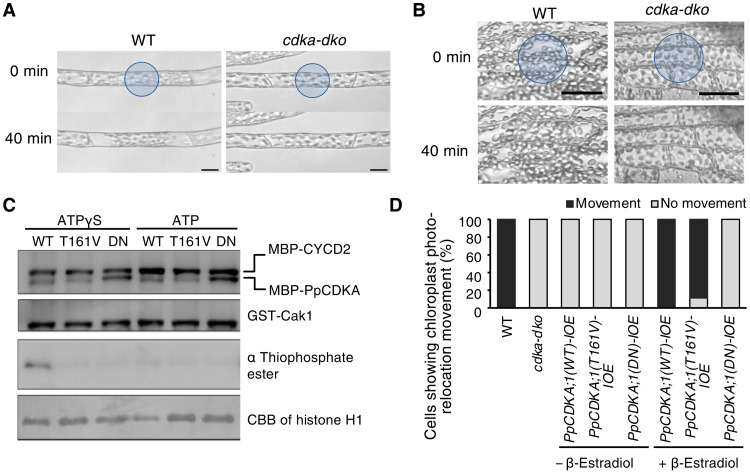
PpCDKA kinase activity requires cell cycle–independent chloroplast photo-relocation. (**A** and **B**) BL-induced chloroplast accumulation response is lost in *cdka-dko* protonemata (A) and gametophores (B). Blue circles indicate the position of the BL microbeam (4.8 μmol m^−2^ s^−1^). Scale bars, 20 μm (A) or 40 μm (B). (**C**) In vitro kinase assay of PpCDKA. Recombinant MBP-PpCDKA;1(WT), MBP-PpCDKA;1 (T161V), or MBP-PpCDKA;1 (DN), along with GST-Cak1 and MBP-CYCD2, was mixed with histone H1 as substrate. An anti-thiophosphate ester antibody was used to detect thiophosphate alkylated histone H1 in the presence of ATPγS. Coomassie brilliant blue (CBB) staining was used to confirm equal loading of histone H1. (**D**) Percentage of cells showing chloroplast photo-relocation in response to a microbeam of BL [WT, *cdka-dko*, *n* = 5 each; *PpCDKA;1*(*WT*)-*IOE*, *PpCDKA;1(T161V)-IOE*, *PpCDKA;1(DN)-IOE*, *n* = 9 each].

Plant cells also adjust the position of their chloroplasts to maximize light capture under weak light and to prevent damage to structures from strong light (*22*). Chloroplast relocation in response to weak BL or RL was lost in *cdka-dko* mutants (Fig. 2A; fig. S3, E and F; and movies S1 and S2). However, the two single mutants *ppcdka;1* and *ppcdka;2* showed the same light responses as the WT (fig. S3, G to K). Therefore, we concluded that CDKA;1 and CDKA;2 act redundantly to control multiple light responses: phototropism, polarotropism, and chloroplast relocation.

### PpCDKAs show cell cycle–independent light responses

Although phototropism, polarotropism, and light-mediated chloroplast relocation are thought to be independent of the cell cycle, the involvement of PpCDKAs in these processes prompted us to specifically test this hypothesis in *P. patens*. To this end, we blocked cell cycle progression pharmacologically with aphidicolin, an inhibitor of DNA replication that specifically arrests cells in early S phase (*16*, *23*). In mock-treated WT protonemata, we observed continuous cell division in apical cells, but aphidicolin treatment completely prevented this, resulting in a single long apical cell. However, light-mediated chloroplast relocation movement and a phototropic response were still evident, suggesting that these light responses can be uncoupled from the cell cycle (fig. S4A). *PpCDKA;1* and *PpCDKA;2* are expressed in both dividing (e.g., protonemal apical) and nondividing cells (e.g., basal protonemal tissues and fully differentiated gametophytic leaves) (*16*) with abundant chloroplasts. Nondividing cells showed light-mediated chloroplast relocation in the WT but not in *cdka-dko* mutants (Fig. 2, A and B, and fig. S4, B and C), supporting our hypothesis that PpCDKAs control chloroplast relocation and phototropic responses independent of their role in the cell cycle.

We next assessed the requirement for CDKA kinase activity in mediating light responses by generating two PpCDKA variants: a dominant-negative form [CDKA(DN)] carrying an Asp-to-Asn substitution at residue 146, which results in loss of kinase activity and cell cycle arrest by preventing Mg^2+^–adenosine 5′-triphosphate (ATP) phosphate binding (*16*, *24*), and a Thr-to-Val substitution at residue 161 (T161V) within the CDK T-loop that is normally phosphorylated to activate the kinase during cell cycle progression (*25*, *26*). Both variants lacked kinase activity, as demonstrated by their inability to phosphorylate histone H1, a common substrate for CDK1, in an in vitro kinase assay with recombinant proteins (Fig. 2C). We then transformed the *cdka-dko* mutant with constructs driving expression of *PpCDKA;1(WT)*, *PpCDKA;1(T161V)*, and *PpCDKA;1(DN)*, each tagged with the Dendra2 fluorescent protein, under the control of a β-estradiol–inducible promoter [lines named *PpCDKA;1(WT)-IOE*, *PpCDKA;1(T161V)-IOE*, and *PpCDKA;1(DN)-IOE*, respectively] (*27*). The expression of all three variants was induced to similar levels by β-estradiol (fig. S5A), allowing a direct comparison of their phenotypes. We evaluated these PpCDKA variants and the role of CDKA kinase activity during the cell cycle. First, because environmental conditions such as nutrient availability affect cell cycle progression (*28*), we grew WT and *cdka-dko* plants under nitrogen limitation, which revealed that mutant protonemal cells are defective in cell cycle progression, as evidenced by the greater 1C DNA content seen in *cdka-dko* plants relative to the WT (fig. S5B). Even under these nitrogen-limited conditions, induction of *PpCDKA;1(WT)* expression with β-estradiol fully rescued the cell cycle defects seen in *cdka-dko* plants, as demonstrated by the predominant 2C cell population (fig. S5B). By contrast, induction of either *PpCDKA;1(T161V)* or *PpCDKA;1(DN)* expression did not rescue these cell cycle defects, as reflected by the predominant 1C peak (fig. S5B). Thus, PpCDKA kinase activity is essential for cell cycle progression.

Next, we characterized the light responses of the *P. patens* lines expressing WT *PpCDKA* or each point mutant. As with the cell cycle, expression of *PpCDKA;1(WT)*, but not *PpCDKA;1(DN)*, rescued light-induced tropism and chloroplast relocation under both nitrogen-replete and nitrogen-limited conditions (Fig. 2D and fig. S5, C to E). Unexpectedly, induction of *PpCDKA;1(T161V)* expression by β-estradiol also rescued light-induced tropism and chloroplast relocation in response to RL and BL (Fig. 2D and fig. S5, C to E). Although we were not able to detect kinase activity for PpCDKA;1(T161V) or PpCDKA;1(DN) in our in vitro assay, we postulate that PpCDKA;1(T161V) may have higher residual kinase activity than PpCDKA;1(DN), as previously reported in tobacco (*Nicotiana tabacum*) BY-2 cells (*26*). We thus propose that full PpCDKA kinase activity is required for cell cycle control, whereas lower kinase activity is sufficient for the light responses in *P. patens* described here.

### PpCDKAs influence the cytoskeleton organization in response to light

**Fig. 3.**
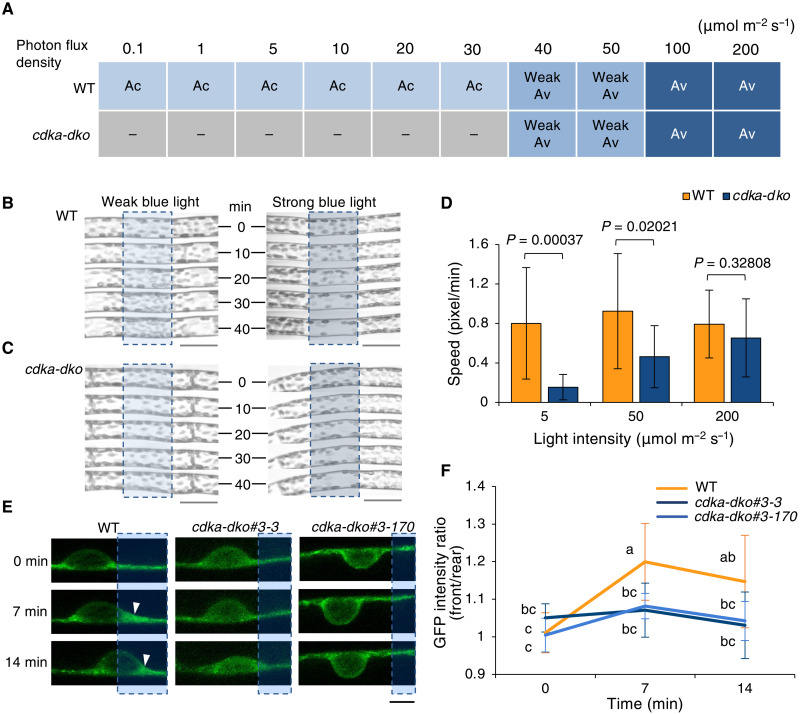
PpCDKAs control chloroplast accumulation via formation of chloroplast actin (cp-actin). (**A**) Chloroplast photo-relocation under various intensities of BL. Chloroplast accumulation (Ac) and avoidance (Av) responses were scored. Cells were illuminated for 40 min with the microbeam. “–“ indicates no chloroplast movement. (**B** and **C**) Chloroplast photo-relocation by a weak (5 μmol m^−2^ s^−1^) or strong (200 μmol m^−2^ s^−1^) BL microbeam in the WT (B) and *cdka-dko* mutant (C). Dashed boxes indicate the region irradiated. Scale bars, 40 μm. The experiment was performed three times independently. (**D**) Speed of chloroplast movement under different light intensities. Data are shown as means ± SD from at least 10 chloroplasts. *P* values are from a two-tailed Welch’s *t* test. (**E**) Representative images of actin around chloroplasts. White arrowheads indicate the brighter GFP foci of cp-actin in GFP-mTalin plants. Dashed boxes indicate the illuminated area; the images were taken just before (0 min) and 7 and 14 min after the start of illumination. Scale bar, 5 μm. (**F**) Changes in the ratio of fluorescence for cp-actin between the front (near the illuminated area) and back of chloroplasts. Data are shown as means ± SD from 7 to 13 chloroplasts. Different letters indicate statistically significant differences by Tukey’s post hoc test.

To understand how CDKA controls chloroplast photo-relocation, we first asked whether light perception itself might be impaired in *cdka-dko* plants. Accordingly, we characterized chloroplast relocation over a wide range of BL or RL intensities. WT chloroplasts accumulated within the illuminated area when exposed to weak BL or RL but moved out of this area at higher fluences, consistent with a light avoidance response (*29*, *30*). WT chloroplasts transitioned from accumulation to avoidance when BL intensity reached about 40 μmol m^−2^ s^−1^ (Fig. 3A). In sharp contrast, *cdka-dko* chloroplasts exhibited no accumulation response, although they did initiate an avoidance response at the same threshold of light intensity as in the WT (Fig. 3, A to C). Quantitative analysis consistently revealed the loss of accumulation response in the double mutant, exhibiting significant reduction in the velocity of chloroplast movement in the *cdka-dko* mutant compared to the WT under low BL intensity, but not during avoidance at higher fluences (Fig. 3D). These results suggested that (i) light perception itself is unaffected in the mutant and (ii) PpCDKA function is specifically required during the accumulation response of light-induced chloroplast relocation under low light intensities.

As the perception of light signals was unaffected in *cdka-dko* mutants, we reasoned that PpCDKA activity might target other downstream effectors, such as the cytoskeleton, which is associated with both phototropism and light-induced chloroplast movement (*29*, *31*). To test this idea, we perturbed the cytoskeleton in WT protonemal cells with oryzalin (a microtubule depolymerizer), TIBA (2,3,5-triiodobenzoic acid; an actin stabilizer), or latrunculin B (Lat B; an actin polymerization inhibitor). Phototropic responses in treated WT cells phenocopied the *cdka-dko* phenotype of unidirectional growth, although to a milder extent in Lat B–treated cells, whereas *cdka-dko* mutants showed little response to any pharmacological agents (fig. S6, A to C). These results suggested that phototropic defects in *cdka-dko* mutants may be due to compromised microtubule and actin cytoskeletons. During light-induced movement of chloroplasts, chloroplast actin (cp-actin) foci have been reported to form at the periphery of chloroplasts to drive their relocation (*32*). When we examined the distribution of cp-actin in the WT and *cdka-dko* mutants using the actin-binding protein mTalin fused to green fluorescent protein (GFP), cp-actin foci did not form at the periphery of *cdka-dko* chloroplasts (Fig. 3, E and F). These data suggested that under weak light intensities, PpCDKAs may directly or indirectly influence the cytoskeleton organization in the cytosol near chloroplasts to direct chloroplast accumulation (*32*) and, at the tip of apical protonemal cells, to modulate phototropic responses (*33*). Time-gated confocal microscopy of fluorescently tagged PpCDKA;1-Dendra2 and PpCDKA;2-Dendra2 proteins in transgenic *P. patens* lines revealed that both proteins localize to the nucleus and the cytosol (fig. S6D). Thus, PpCDKAs likely affect light responses by acting on the cytoskeleton in the cytosol.

### Conservation of CDKA function in land plants

**Fig. 4.**
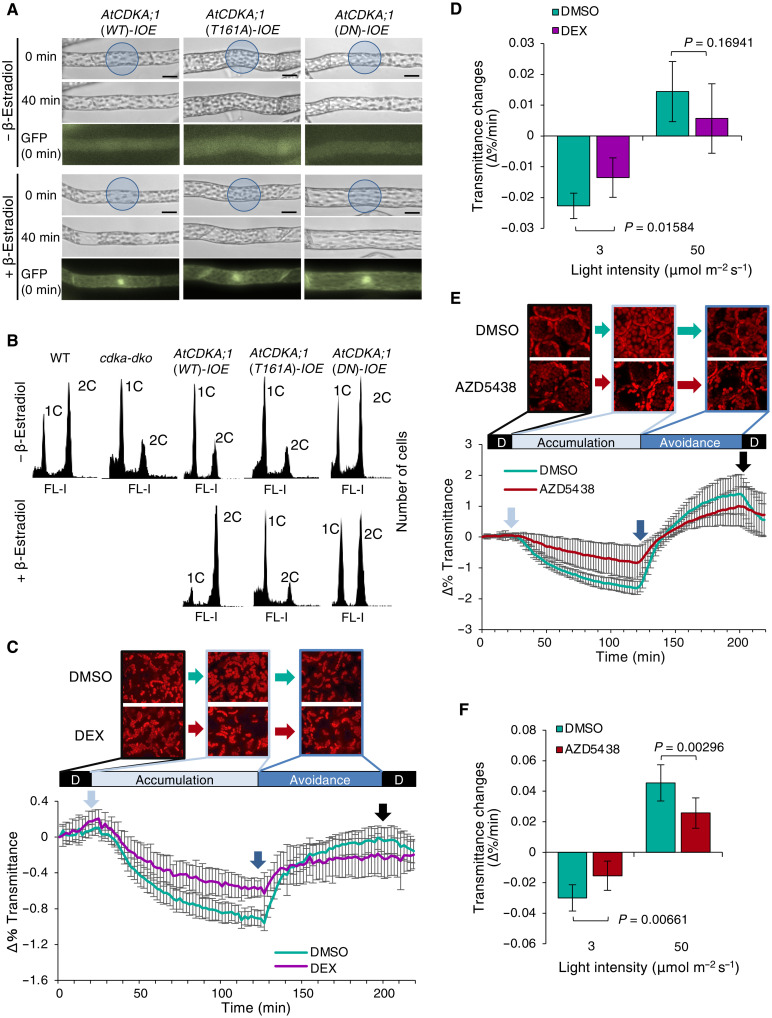
AtCDKA;1 participates in the chloroplast accumulation response in *P. patens* and *Arabidopsis*. (**A**) *Arabidopsis* CDKA;1 rescues the chloroplast accumulation defect of *P. patens cdka-dko* mutants. Scale bars, 20 μm. Successful induction of transgene expression by 1 μM β-estradiol was confirmed by GFP fluorescence (bottom). (**B**) *AtCDKA;1(WT)*, but not *AtCDKA;1*(*T161A*) or *AtCDKA;1*(*DN*), rescues the cell cycle defects in *cdka-dko* mutants. Ploidy profiles from different genotypes were determined from plants grown on nitrogen-deficient medium for 5 days. (**C** to **F**) Analysis of chloroplast movement, as determined by leaf transmittance. Leaves were treated with 0.1% dimethyl sulfoxide (DMSO) or 20 μM dexamethasone (DEX) for *AtCDKA;1*(*DN*)-*IOE* transgenic *Arabidopsis* plants (C and D) or with 0.1% DMSO or 10 μM AZD5438 for the WT (E and F). (C and E) Top: Representative photographs of the surface of mesophyll cells from dark-adapted leaves (D) and leaves illuminated with weak BL (accumulation) or strong BL (avoidance). The percentage of change in transmittance is shown for *AtCDKA;1*(*DN*)-*IOE* plants (C, bottom) and the WT (E, bottom). Light blue arrow, illumination with 3 μmol m^−2^ s^−1^; dark blue arrow, illumination with 50 μmol m^−2^ s^−1^; black arrow, return to darkness. (D and F) Mean transmittance change, in percent. Data are shown as means ± SD of three leaves. *P* values are from a two-tailed Welch’s *t* test.

We next explored the conservation of CDKA function across land plants. The genome of the flowering plant Arabidopsis (*Arabidopsis thaliana*) encodes a single PSTAIRE-CDK, AtCDKA;1 (*6*). We introduced *AtCDKA;1* into the *cdka-dko* background, as well as two variant forms of the kinase: *AtCDKA;1(DN)* and *AtCDKA;1(T161A)* (*26*). The light-induced tropism and chloroplast movement defects characteristic of *cdka-dko* mutants were rescued by the induction of *AtCDKA;1(WT)* and *AtCDKA;1(T161A)* expression, but not *AtCDKA;1(DN)* expression (Fig. 4A and fig. S7, A and B). However, only the expression of *AtCDKA;1(WT)* rescued the cell cycle defects of *cdka-dko* mutants (Fig. 4B). These results demonstrated the conservation of function between PpCDKAs and AtCDKA;1 for the regulation of the cell cycle and light responses. Notably, both *PpCDKA;1(T161V)* and *AtCDKA;1(T161A)* retained sufficient function to fulfill the requirement of CDKA kinase activity in the context of light responses, but not the cell cycle.

We next explored the role of AtCDKA;1 in light responses in *Arabidopsis*. As the *Arabidopsis cdka;1* mutant displays a severe growth defect that prevented any examination of its light responses, we investigated chloroplast movement in a transgenic *Arabidopsis* line in which the dominant-negative variant *AtCDKA;1(DN)* was placed under the control of a dexamethasone (DEX)–dependent transgene expression system, by exposing seedlings to light conditions resulting in chloroplast accumulation and then avoidance (weak or strong BL, respectively). We determined that chloroplast movement is compromised during the accumulation response but not the avoidance response (Fig. 4, C and D, and fig. S7C). We also treated *Arabidopsis* rosette leaves with AZD5438, a specific CDK1 inhibitor (*34*). Treatment with AZD5438 strongly compromised both the accumulation and avoidance responses of chloroplasts (Fig. 4, E and F). Such reduced chloroplast movement was unlikely to stem from AZD5438 cytotoxicity, as the photosystem II activity parameter *F*_v_*/F*_m_ showed comparable values in mock- and AZD5438-treated chloroplasts (fig. S7D). Thus, AtCDKA;1, like PpCDKAs, appeared to control chloroplast accumulation in *Arabidopsis*. We then tested the phototropic response of hypocotyls from *Arabidopsis* seedlings induced to express *AtCDKA;1*(*DN*) with DEX: Their hypocotyl curvature decreased relative to that of seedlings treated with dimethyl sulfoxide (DMSO) only, irrespective of light intensity (fig. S7, E and F). Thus, although our results on AtCDKA;1 function mostly stem from heterologous *AtCDKA;1* expression in the moss *cdka-dko* mutants, the *AtCDKA;1* dominant-negative form expression in *Arabidopsis*, or the inhibitor treatment in *Arabidopsis*, we conclude that CDKAs can have conserved function in light-induced chloroplast movement and phototropic responses in both *P. patens* and *Arabidopsis*.

## DISCUSSION

In addition to their expected roles in cell cycle progression in the nucleus, PSTAIRE-type CDKAs in the moss *P. patens* also modulate light responses, likely by regulating cytoskeleton activity in the cytosol (fig. S8). We propose that this function is evolutionarily conserved across land plants. The natural light environment fluctuates dynamically, and light signaling cascades and responses like phototropism and light-induced chloroplast movement should rapidly respond in kind. It is thus tempting to speculate that the bistable switch of the CDK-cyclin complex, which is key to cell cycle progression (*35*), may have been co-opted to regulate light responses in land plants. To elucidate a mechanistic link between the CDKA-cyclin complex and cytoskeletal organization in cytosol, identifying a cyclin partner for CDKA and its substrate is an important next step.

Tropisms exhibited by phototropism or polarotropism and chloroplast movement must preserve directional information of light signals; therefore, signal transduction cascades of light-induced tropism and chloroplast movement are likely within cytoplasmic signaling routes, initiated close to the plasma membrane or in the cytoplasm. By contrast, if the signal transduction cascades pass through the nucleus and gene expression, directional information of light signals will be lost (*22*, *36*, *37*). Although cytoplasmic activities for the RL photoreceptor phytochrome (phy) have been described in many experimental systems, including RL-induced chloroplast reorientation in the green alga *Mougeotia* (*38*), polarotropism and phototropism of protonemata in ferns (*39*) and mosses (*40*, *41*), and cytoplasmic motility in epidermal cell of the aquatic angiosperm *Vallisneria gigantea* (*42*), most studies on phy have focused on the transcriptional control of *PHY* genes (*43*). In *P. patens*, the receptors that provide directional information on RL and BL are phy and phototropin (phot), respectively, whereas *Arabidopsis* phot receptors, generally associated with the plasma membrane, perceive BL signals to induce chloroplast movement and phototropism (*22*, *36*, *37*). In addition, a small pool of phy interacts with phot at the plasma membrane in *P. patens* (*40*), thereby providing directional information within the cytoplasm. Thus, the light signal transduction cascades initiated from either photoreceptor evoke changes in cytoskeletal microtubules, actin filaments, and cp-actin, playing an important role in phototropism, polarotropism, and chloroplast movement (*22*, *29*, *31*). Several cytoplasmic components that link photoreceptors to the cytoskeleton have been identified for their role in the chloroplast movement response, including KAC (kinesin-like protein for actin-based chloroplast movement), THRUMIN1, and CHUP1 (chloroplast unusual positioning1) in *P. patens* and *Arabidopsis* (*22*), but not for light-mediated tropisms. We propose that both PpCDKAs and AtCDKA;1 likely constitute a novel signaling regulator in *P. patens* and *Arabidopsis*, respectively, connecting photoreceptors to cytoskeletons; the transduction of the directional signal may be derived from the cytoplasmic activity of CDKAs (fig. S6D).

On the other hand, we noticed that the effect of AtCDKA;1 on hypocotyl phototropism was modest (fig. S7, E and F). The expression of *AtCDKA;1(DN)* was sufficiently induced in *Arabidopsis* hypocotyls after treatment with DEX (fig. S7E), making expression levels an unlikely culprit. One possible explanation for this weak phenotype might be the single-cell scale of light-induced chloroplast movement in *P. patens* and *Arabidopsis* (*22*) and phototropism and polarotropism of protonemata in *P. patens* (*21*), in contrast to the integrated response of a three-dimensional tissue for hypocotyl phototropism, which is caused by differential growth between the irradiated and shaded sides due to asymmetric distribution of the phytohormone auxin (*44*). Such differences in the light responses of single cells or three-dimensional tissues may thus provide a rationale for the mild effects of AtCDKA;1 on hypocotyl phototropism. In a nonmutually exclusive alternative, redundant pathways for hypocotyl phototropism have been suggested in *Arabidopsis* (*45*), in which AtCDKA;1 may participate. Further studies on AtCDKA;1 function in *Arabidopsis* will extend our understanding of the mechanisms underlying light responses in land plants.

In animals, the PSTAIRE-type CDKs CDK1 and CDK2 have been recently shown to be involved in non–cell cycle functions including rapid immune system responses, neutrophil migration, and motile ciliogenesis, although genetic experiments in which the PSTAIRE-type CDKs are knocked out should be done to corroborate these results (*10*–*13*). Our study expands on these findings by adding PSTAIRE-type CDKs from the plant kingdom to this list, providing unambiguous genetic evidence for non–cell cycle functions of PSTAIRE-type CDKs by using a double knockout mutant in *P. patens*. Although PSTAIRE-type CDKs have been considered critical players specifically for cell cycle regulation, mutant phenotypes that were thought to be caused by a cell cycle defect might be caused by an overlooked defect in environmental responses. Various proteins with roles outside of cell cycle regulation have been reported to be phosphorylated by yeast CDC28 and mouse CDK1, but few of these candidate substrates have been studied in detail, likely due to lack of evidence that they are true targets for CDC28 or CDK1 phosphorylation (*46*–*48*). We therefore hypothesize that PSTAIRE-type CDKs are fundamental kinases with essential roles in diverse processes besides cell cycle regulation across all eukaryotes. PSTAIRE-type CDKAs may interact with different activators and/or cyclins, show distinct kinase activity, and be regulated spatially and temporally to support various cellular processes throughout the cell life cycle. In plants, this previously unidentified function of CDKA is important for environmental adaptation; in animals, the new proposed function for CDK1 may also explain impaired cellular responses to the environment and, ultimately, diseases. Thus, our findings should motivate research on these CDK-interacting partner candidates as true substrates and could uncover a range of important functions for PSTAIRE-type CDKs during plant and animal growth and environmental responses. Further study of the molecular mechanisms behind CDKA regulation during light responses will not only deepen our understanding of plant adaptations to their dynamic light environments but also reveal how eukaryotes have deployed PSTAIRE-type CDKs during evolution to respond to their surroundings.

## MATERIALS AND METHODS

### Plant materials and culture conditions

*Physcomitrium* (*Physcomitrella*) *patens* Bruch & Schimp subsp. *patens* was used as the WT (*49*). Moss tissues were cultured in BCDATG or BCDAT agar medium (*49*) and grown under constant white light conditions (∼50 μmol m^−2^ s^−1^) provided by 40-W white fluorescent light bulbs (FLR40S-EX-N/M/36; Mitsubishi-Osram) at 22° to 25°C. Polyethylene glycol–mediated transformation of *P. patens* was performed as described previously (*49*). *Arabidopsis* (*A. thaliana*) accession Columbia-0 (Col-0) was grown in plastic dishes with B5 (Nihon Pharmaceutical) agar medium or in pots containing peat moss and vermiculite in a 1:6 ratio in constant white light (∼75 μmol m^−2^ s^−1^) with 40-W white fluorescent light bulbs (FLR40S-EX-N/M/36; Mitsubishi-Osram) at 23°C.

### Protein extraction and immunoblot analysis

Protein extracts prepared from protonemata were analyzed by SDS–polyacrylamide gel electrophoresis (PAGE) as described previously (*16*). For immunoblot analysis, proteins were transferred to an Immobilon-P membrane (Millipore) and detected with a monoclonal anti-PSTAIRE antibody (P7962; Sigma-Aldrich), a polyclonal anti-dendra2 antibody (AB821; Evrogen), or a monoclonal anti–α-tubulin antibody (DM1A; Sigma-Aldrich) as primary antibodies. A horseradish peroxidase (HRP)–conjugated anti-rabbit secondary antibody (Cytiva) was used at a 1:5000 dilution for visualization with ECL Plus (Cytiva); the results were documented using the LAS-3000 system (Cytiva).

### Flow cytometry analysis of DNA content

Protonemal tissues of *P. patens* were subcultured for 5 to 8 days on agar medium overlaid with cellophane in constant white light (∼50 μmol m^−2^ s^−1^) before collection. To induce gametophores, moss tissue was grown in BCDAT medium without cellophane for 1 month in white light (∼50 μmol m^−2^ s^−1^); the gametophores were then collected from the medium, and the rhizoids were removed. The resulting materials were chopped with a razor blade in 200 μl of extraction buffer [200 mM tris-HCl (pH 7.5), 4 mM MgCl_2_, 0.5% (v/v) Triton X-100] in a 9-cm petri dish and incubated for 2 min at room temperature. Then, another 200 μl of extraction buffer was applied to the chopped tissue. The extracted nuclei were filtered through a 30-μm mesh nylon sieve (CellTrics). The homogenized sample was then stained with 1 ml of 4′,6-diamidino-2-phenylindole dihydrochloride (0.5 μg/ml) (Wako) in phosphate-buffered saline [130 mM NaCl, 7 mM Na_2_HPO_4_, 3 mM NaH_2_PO_4_ (pH 7.4)] at room temperature for 10 min in the dark. The DNA content was determined by fluorometry (Ploidy Analyzer, Partec).

### Plasmid construction and yeast transformation

The primers used for plasmid construction are listed in table S1. The GenBank accession numbers for the genes whose coding sequences were cloned are AJ515321 (*PpCDKA;1*), AB547329 (*PpCDKA;2*), XM_024543629 (*PpCDKB;1*), and XM_024511534 (*PpCDKB;2*). To generate pENTR-*PpCDKA;1*, pENTR-*PpCDKA;2*, pENTR-*PpCDKB;1*, and pENTR-*PpCDKB;2*, the open reading frames of *PpCDKA;1*, *PpCDKA;2*, *PpCDKB;1*, and *PpCDKB;2* were amplified by polymerase chain reaction (PCR) using KOD plus high fidelity polymerase (Toyobo) and cloned into the pENTR/D-TOPO vector (Invitrogen). To generate pGADT7-*PpCDKA;1*, pGADT7-*PpCDKA;2*, pGADT7-*PpCDKB;1*, and pGADT7-*PpCDKB;2*, the vectors pENTR-*PpCDKA;1*, pENTR-*PpCDKA;2*, pENTR-*PpCDKB;1*, and pENTR-*PpCDKB;2* were recombined into the destination vector pGADT7-DEST, obtained from the Arabidopsis Biological Resource Center (ABRC), by LR recombination (Invitrogen).

To perform complementation tests in the *S. cerevisiae cdc28-4* temperature-sensitive mutant, the vectors pGADT7-*PpCDKA;1*, pGADT7-*PpCDKA;2*, pGADT7-*PpCDKB;1*, and pGADT7-*PpCDKB;2*, as well as the empty destination vector pGADT7, were transformed into the *cdc28-4* mutant (provided by M. Umeda, Nara Institute of Science and Technology) using the Frozen-EZ Yeast Transformation II kit (ZYMO RESEARCH). The resulting transformants were plated on synthetic defined agar medium lacking Leu (SD −Leu) and grown for 8 days at 22°C in the dark for selection. Five independent colonies from each transformation were inoculated in 100 μl of liquid YPD medium and cultivated at 25°C with shaking at 200 rpm overnight. The cells were pelleted by centrifugation at 1300*g* for 5 min at room temperature, washed, and resuspended in sterile water. The cells were then plated on SD −Leu agar medium and grown for 10 days at 22°C (permissive temperature) or 37°C (restrictive temperature). Complementation of the mutant was assessed by growth at 37°C. Results were consistent across all colonies for each transformation.

### Plasmid construction for *P. patens* transformation

*P. patens cdka-dko* knockout mutants were obtained by homologous recombination (*16*). The structure of the *PpCDKA;1* and *PpCDKA;2* loci is shown in fig. S1A. To generate the *PpCDKA;1-ko* plasmid, a 1184–base pair (bp) fragment upstream and an 835-bp fragment downstream of *PpCDKA;1* were amplified from genomic DNA by PCR and cloned into pTN182 (accession number: AB267706). For *PpCDKA;2-ko*, a 1163-bp fragment upstream and a 1158-bp fragment downstream of *PpCDKA;2* were amplified from genomic DNA by PCR and cloned into pTN186 (accession number: AB542059). The resulting constructs were used for *P. patens* transformation to obtain the single knockout mutants *ppcdka;1* and *ppcdka;2.* To generate the *cdka-dko* double knockout mutants, the *PpCDKA;1-ko* fragment was serially transformed into the *ppcdka;2* single knockout mutant. Single insertion lines were screened by Southern blot analysis as described (*16*).

To remove the *aphIV* and *nptII* cassettes from the *cdka-dko* lines, a construct bearing *35S:Cre* (*50*) (provided by F. Nogué, Institut National de la Recherche Agronomique) was transformed into *cdka-dko* line #5 to transiently express the Cre recombinase to remove *aphIV* and *nptII*. The resulting *aphIV-* and *nptII*-free *cdka-dko* line was confirmed by its inability to grow in medium containing hygromycin and G418, following a previously described method (*51*). The *aphIV-* and *nptII*-free *cdka-dko* line has a phenotype identical to that of the original *cdka-dko* line #5 for the light responses examined.

The mutation T161V was introduced into *PpCDKA;1* through overlapping PCR. Two fragments were PCR amplified using pENTR-*PpCDKA;1* as template and mixed in a 1:1 ratio to serve as template for a second round of PCR. The second PCR amplicon was then cloned into pENTR to generate pENTR*-PpCDKA;1(T161V).*

To generate steroid-inducible constructs, the vector pPGX8 (accession number: AB537482) was used (*27*), which harbors the steroid-inducible *XVE* cassette and the *aphIV* gene conferring resistance to hygromycin. To generate pPGX8-*PpCDKA;1*(*WT*)*-dendra2*, pPGX8-*PpCDKA;1*(*T161V*)*-dendra2*, and pPGX8-*PpCDKA;1*(*DN*)*-dendra2*, the open reading frame of WT and mutant versions of *CDKA;1* (*WT*, *T161V*, and *DN*) lacking the stop codon was fused to a PCR fragment encoding the Dendra2 tag by overlapping PCR. Briefly, PCR fragments for *PpCDKA;1*(*WT*), *PpCDKA;1(T161V*), and *PpCDKA;1*(*DN*) were PCR amplified from pENTR-*PpCDKA;1*(*WT*), pENTR-*PpCDKA;1*(*T161V*), and pENTR-*PpCDKA;1*(*DN*)*-3HA* (*16*) as templates, respectively. The Dendra2 sequence was PCR amplified using *pT1OG-dendra2* (*52*) as template. The two PCR fragments were mixed in a 1:1 ratio and used as template for a second PCR; the resulting amplicons of which were cloned into the pENTR/D-TOPO vector to generate the constructs pENTR-*PpCDKA;1*(*WT*)*-dendra2*, pENTR-*PpCDKA;1*(*T161V*)*-dendra2*, and pENTR-*PpCDKA;1*(*DN*)*-dendra2*. Last, pENTR-*PpCDKA;1*(*WT*), pENTR-*PpCDKA;1*(*WT*)*-dendra2*, pENTR-*PpCDKA;1*(*T161V*)*-dendra2*, and pENTR-*PpCDKA;1*(*DN*)*-dendra2* were recombined into the destination vector pPGX8 by LR recombination. The final constructs were digested with *Sse*8387I (Takara) for gene targeting and introduced into the *aphIV-* and *nptII*-free *cdka-dko* line #5.

### Plasmid construction for cross-species complementation

To generate *AtCDKA;1*(*WT*)*-GFP*, *AtCDKA;1*(*DN*)*-GFP*, and *AtCDKA;1*(*T161A*)*-GFP* constructs, the plasmids pGpro*AtCDKA;1*(*WT*)*-GFP*, pGpro*AtCDKA;1*(*DN*)*-GFP*, and pGpro*AtCDKA;1*(*T161A*)*-GFP* (*26*) were used as PCR templates. The resulting PCR products were then cloned into the pENTR/D-TOPO vector to generate pENTR-*AtCDKA;1*(*WT*)*-GFP*, pENTR-*AtCDKA;1*(*DN*)*-GFP*, and pENTR-*AtCDKA;1*(*T161A*)*-GFP*, which was followed by LR recombination into the destination vector pPGX8 to generate pGX8-*AtCDKA;1*(*WT*)*-GFP*, pGX8-*AtCDKA;1*(*DN*)*-GFP*, and pGX8-*AtCDKA;1*(*T161A*)*-GFP*. The final constructs were digested with *Sse*8387I for gene targeting and introduced into the *aphIV*- and *nptII*-free *cdka-dko* line #5.

### Plasmid construction and *Arabidopsis* transformation

To generate pOpON-*AtCDKA;1*(*DN*)*-GFP*, pENTR-*AtCDKA;1*(*DN*)*-GFP* was recombined with the destination vector pOpON (provided by A. Tanaka, Hokkaido University) by LR recombination. The resulting pOpON-*AtCDKA;1*(*DN*)*-GFP* construct was transformed into *Arabidopsis* accession Col-0 by the floral dip method (*53*). T_1_ seeds were sown on B5 medium containing kanamycin (50 μg/ml) for selection. The presence of the transgene was confirmed by assessing GFP fluorescence in seedlings grown on B5 medium containing 1 μM DEX by fluorescence microscopy and by segregation analysis for kanamycin resistance.

### Gravitropism, phototropism, and polarotropism assays in *P. patens* protonemata

For the analysis of gravitropism, light-grown plants were cultured along the surface of BCDATG agar medium plates placed vertically in the dark. For the analysis of RL- and BL-induced phototropism, light-grown plants were cultured on the surface of BCDATG agar medium in 13 × 13 cm square dishes (NIPRO) sealed with surgical tape (3MM). Three sides of the plate were sealed with black vinyl tape (Nichiban) and wrapped with sheets of black paper and aluminum foil. Plants were then continuously exposed to unilateral light through the uncovered and unsealed side of the plate with red light-emitting diodes (LEDs) (λ_max_ = 660 ± 20 nm; Stick-mR, Tokyo Rikakikai, Tokyo, Japan) or blue LEDs (λ_max_ = 470 ± 30 nm; Stick-mB). The phototropic response of protonemata was determined 7 to 14 days after onset of continuous illumination using a stereomicroscope (SZX12; Olympus) equipped with a digital camera (DMC-G2; Panasonic). For the analysis of polarotropism, protonematal tissues were cultured on the surface of BCDATG agar medium plates and continuously illuminated with polarized light from above. Light was provided by 40-W white fluorescent tubes (FLR40S-EX-N/M/36) unless stated otherwise. The light passed through a 3-mm-thick red or blue acrylic plastic filter (Mitsubishi) or was provided by red/blue LEDs and a polarizer (HN32; Sumitomo 3 M) into light-proof boxes. Because the plastic petri dishes depolarized the polarized light, the petri dish lids were replaced by glass dishes. The polarotropic response of protonemal filaments was determined using images taken with a stereomicroscope (SZX12) equipped with a digital camera (DMC-G2). For quantification of phototropism and polarotropism, protonemata longer than 0.5 mm were used for measurements, and angles were determined at the point of growth axis 0.3 mm from the base to the tip of the protonemal filaments.

### Light-induced chloroplast relocation in protonemata and gametophores

Microbeam illumination was performed on a custom-made microbeam irradiator from an inverted microscope (ECLIPSE Ti2, Nikon) equipped with an epifluorescence unit. To produce BL, a halogen lamp was coupled to an interference filter with a 445-nm peak and a half bandwidth of 49.5 nm (Semrock). The dichroic mirror was replaced with a half mirror (OPL-HM50/50-25.2x35.6). Light intensity was measured using an optometer (UDT S370, GAMMA SCIENTIFIC). The formula for calculating photon flux density (PFD) is given as

$$\text{PFD}\;(\mu \text{mol}/m^{2}/s)=\frac{\text{irradiance}\;(W/m^{2})\times \lambda \;(m)\times 10^{6}}{N_{A}\;({\text{mol}}^{-1})\times h\;(J・s)\times c\;(m/s)}$$

where λ is the wavelength, *N*_A_ (6.022 × 10^23^ mol^−1^) is the Avogadro constant, *h* (6.626 × 10^−34^ J・s) is the Planck constant, and *c* (3.0 × 10^8^ m/s) is the speed of light.

For measuring chloroplast movement using a microbeam, protonemata were grown in BCDATG medium solidified with 0.5% (w/v) gellan gum (Nacalai Tesque) in a glass-bottom dish (Iwaki) and were precultured in RL irradiated from above at 0.5 μmol m^−2^ s^−1^ for 1 week. Chloroplast positions were monitored every 2 min for 40 min. The intensity of the blue microbeam was set to 1 μmol m^−2^ s^−1^ to induce a chloroplast accumulation response. To compare the speed of the chloroplast movement, the values for 16 to 20 min after irradiation were used. Data were analyzed with ImageJ (http://rsb.info.nih.gov/ij/). Alternatively, movement rate was determined following a previous protocol (*54*). Briefly, subapical cells were used for illumination for 30 min with the BL microbeam (10 μm wide) at a fluence rate of 0.4 μmol m^−2^ s^−1^ and were photographed every minute during irradiation. For each line, chloroplast movement was examined from three protonemata. Individual chloroplasts were traced by time-lapse imaging as described previously (*55*). Chloroplast movements were determined by measuring the moving velocity of chloroplasts toward the microbeam-irradiated area in ImageJ.

For the analysis of RL-induced chloroplast movement in protonemata, plants were grown in 3 ml of BCDATG medium solidified with 0.5% (w/v) gellan gum (Wako) in 27-mm glass-bottom dishes (Iwaki). The dishes were then inverted and the plants were grown in weak RL (0.5 μmol m^−2^ s^−1^) from above for 5 days to allow the protonemata to attach to the glass bottom. To analyze unilateral RL-induced chloroplast redistribution (due to chloroplast photo-relocation) in protonemata, the plants were illuminated with unilateral light for 20 hours with the light parallel or perpendicular to the protonemal growth axis. To analyze chloroplast movement induced by polarized RL in protonemata, the plants were illuminated with polarized RL from above for 20 hours with the E-vector perpendicular or parallel to the protonemal growth axis. Unilateral RL and polarized RL were produced by passing light through a 3-mm-thick red (no. 102; Mitsubishi) acrylic plastic filter without (for unilateral RL) or with a polarized filter (for polarized RL) into light-proof boxes. Light was provided by 40-W white fluorescent tubes (FLR40S-EX-N/M/36; Mitsubishi). The distribution of chloroplasts in protonemata was recorded with a microscope (Leica DMLB) equipped with a charge-coupled device digital camera (Cool SNAP, Photometrics).

For the analysis of unilateral RL-induced chloroplast redistribution in gametophores, plants were grown on BCDATG medium solidified with 0.5% (w/v) gellan gum in 27-mm glass-bottom dishes under unilateral RL at 0.5 μmol m^−2^ s^−1^ for more than 3 weeks. Chloroplast distribution in gametophores was recorded with a microscope (Leica DMLB) equipped with a digital camera as described above.

### Generation of *cdka-dko* plants in *GFP-mTalin* lines and cp-actin observation

The *GFP-mTalin* (*56*) transgenic *P. patens* line was provided by A. Kadota (Tokyo Metropolitan University) (*57*). To generate the *cdka-dko* plants in the background of the *GFP-mTalin* line, the 5′ and 3′ flanking regions of *PpCDKA;1* were PCR amplified from *P. patens* genomic DNA with the primers shown in table S1, followed by cloning into the Xho I and Sal I sites (for the 5′ end) and Xba I and Not I sites (for the 3′ end) of the pTN182-hyg plasmid by hot fusion (*58*), where pTN182-hyg was created by replacing the *nptII* cassette with *aphIV* from pTN186. Similarly, the 5′ and 3′ fragments of *PpCDKA;2* were inserted by hot fusion into the Xho I and Hind III sites or into the Xba I and Not I sites, respectively, of the p35S-Zeo plasmid (accession number: EF451822). *GFP-mTalin* transformants for *cdka-dko* deficiency were selected on BCDAT medium containing hygromycin (30 mg/liter) or zeocin (100 mg/liter) and further confirmed by polarotropism defects under polarized light.

To observe cp-actin, a confocal laser scanning microscope was used (Zeiss LSM 510). The objective lens was a Plan-Apochromat 63×/1.4 oil differential interference contrast with a microbeam at 488 nm to excite GFP and chlorophyll autofluorescence. The photobleaching mode was used to induce the chloroplast accumulation response; a weak 458-nm microbeam (10 to 30% output) was used with a ND 16 filter (Zeiss) and a ND 5 filter (OPTO-LINE). Images were recorded at the beginning of the experiment (0 min), after 7 min, and after 14 min.

Fiji (https://imagej.net/Fiji) was used to measure the intensity of the GFP signal. The front and rear halves of each chloroplast toward the microbeam-irradiated region were enclosed in polygons. The area outside of the cell was also measured to provide a background intensity measurement. GFP intensity ratios (front/rear) were calculated as (mean value of front − mean value of background)/(mean value of rear − mean value of background). The *z* slice with the highest intensity ratio was selected at 7 and 14 min, and the *z* slice at 0 min was chosen if it showed chloroplasts of similar size to the one selected at 7 min. Thirteen, seven, and nine chloroplasts were analyzed in *GFP-mTalin* (WT), *cdka-dko GFP-mTalin* #3-3 (*cdka-dko* #3-3), and *cdka-dko GFP-mTalin* #3-170 (*cdka-dko* #3-170) plants, respectively. Statistical analysis was performed using a two-way analysis of variance and Tukey’s post hoc test (significance level: *P* < 0.05).

### Drug treatments

*P. patens* was inoculated at the bottom of a 27-mm glass-bottom dish in 3 ml of BCDATG medium solidified with 0.5% (w/v) gellan gum. Aphidicolin (Wako), oryzalin (Wako), TIBA (Sigma-Aldrich), or Lat B (Wako) dissolved in DMSO as stock solutions was diluted with water and applied to the surface of the growth medium to the final indicated concentrations. For aphidicolin treatment, protonemata were grown in RL for 5 days before addition of aphidicolin at a final concentration of 10 μg/ml [3 μl of a stock (10 mg/ml) into 3 ml of medium] or 0.1% DMSO (3 μl of DMSO into 3 ml of medium). The plants were then illuminated with unilateral RL (0.5 μmol m^−2^ s^−1^) for 22 hours. After induction of the phototropic response, plates were rotated 90°, while unilateral RL was kept at a fixed angle. For phototropic responses in the presence of oryzalin, TIBA, or Lat B, protonemata were grown under unilateral RL (15 μmol m^−2^ s^−1^) for 12 days (oryzalin), 5 days (TIBA), or 7 days (Lat B).

### In vitro kinase assays

To produce the maltose-binding protein (MBP)–PpCDKA fusion protein, the coding sequences of *PpCDKA;1(WT)*, *PpCDKA;1(T161V)*, or *PpCDKA;1(DN)* were cloned into the pMAL-c2X vector (a gift from M. Trujillo, University of Freiburg) as Bam HI–Hind III fragments. Thereafter, the *MBP-PpCDKA;1(WT)*, *MBP-PpCDKA;1(T161V)*, and *MBP-PpCDKA;1(DN)* inserts were PCR amplified from the above pMAL-c2X vectors and subcloned into the pCDFDuet–glutathione *S*-transferase (GST)–Cak1 vector (a gift from A. Schnittger, University of Hamburg) linearized by digestion with Sac I and Hind III to generate a recombinant dual construct that simultaneously produces MBP-PpCDKA;1 and GST-Cak1 fusion proteins (*59*). To produce the recombinant proteins MBP-PpCDKA;1, GST-Cak1, and MBP-PpCYCD;2 (accession number of PpCYCD;2: AB547332) (*16*), the pCDFDuet-MBP-PpCDKA;1-GST-Cak1 and pMAL-c2X-MBP-PpCYCD;2 constructs were introduced into *Escherichia coli* strain BL21 by heat shock cotransformation. Individual colonies were grown in LB medium until cultures reached an optical density at 600 nm (OD_600 nm_) of 0.5 before addition of 0.3 mM isopropyl β-d-1-thiogalactopyranoside (Wako) and further grown overnight at 18°C. To harvest the recombinant protein complex of MBP-PpCDKA-GST-Cak1-MBP-PpCYCD;2, the cell pellet was resuspended in 7 ml of column buffer [200 mM NaCl, 20 mM tris-HCl (pH 7.4), 1 mM EDTA, 1 mM DTT] before sonication for 5 min at 40% output (Branson Sonifier 250) in the presence of lysozyme (1 mg/ml; Wako). Thereafter, 5% (v/v) Triton X-100 was added to the lysed cells, and the lysate was incubated at room temperature for 30 min with constant agitation. Cell debris were separated from the protein extract by centrifugation at 9560*g* for 30 min at 4°C. The recombinant protein complex was purified using amylose resin [New England Biolabs (NEB)] according to the manufacturer’s instructions.

Kinase reactions were performed as described previously (*60*). In brief, histone H1 (NEB, M2501) was used as a generic kinase substrate. Recombinant proteins were incubated in kinase buffer [50 mM Hepes-KOH (pH 7.5), 20 mM MgCl_2_, 5 mM EGTA (pH 8.5), 1 mM DDT, 1× PhosSTOP; Roche], with either 12 mM ATPγS or 12 mM ATP (Sigma-Aldrich). After incubation for 30 min at room temperature, kinase reactions were stopped by the addition of 40 μM EDTA (pH 8.0). To alkylate the recombinant proteins, 1.5 μl of 50 mM *p*-nitrobenzylmesylate (Abcam) was added directly to each 30-μl kinase reaction, and the mixture was incubated for 1 to 2 hours at room temperature. After alkylation, samples were analyzed by electrophoresis on 12.5% SDS-PAGE gels. An anti-thiophosphate ester-specific antibody (Abcam, ab92570; 1:1000 dilution) in tris-buffered saline with 0.05% (v/v) Tween 20 was used as primary antibody. In parallel, MBP-CDKA and MBP-CYCD2 were detected with an anti-MBP antibody (NEB) at a 1:8000 dilution. For detection of GST-Cak1, an anti-GST antibody (Wako) was used at a 1:1000 dilution. An HRP-conjugated anti-rabbit secondary antibody (Cytiva) was used at a 1:5000 dilution for visualization with ECL Plus (Cytiva) and documented using the LAS-3000 system (Cytiva).

### Analysis of chloroplast photo-relocation movement in *Arabidopsis*

AZD5438 (HY-10012, MedChemExpress) and DEX (Nacalai Tesque) were dissolved in DMSO and stored as 10 mM (AZD5438) and 20 or 30 mM (DEX) stock solutions at −20°C until use. Chloroplast photo-relocation movement was analyzed by measuring changes in leaf transmittance, as described previously (*61*, *62*). Briefly, *Arabidopsis* Col-0 seedlings were grown on B5 medium containing 0.8% (w/v) agar in constant white light at 23°C in a growth chamber. Leaves were then detached from 2- to 3-week-old seedlings, and the air within the leaf space was replaced with the drug solution by pushing the solution into the leaf space with a syringe containing the drug solution, AZD5438 or DEX. Then, the leaves were placed and incubated for 1 day in the dark on 1% (w/v) gellan gum in a 96-well clear bottom plate. The leaves in the 96-well plate were kept in the dark for another 20 min and exposed to weak BL (3 μmol m^−2^ s^−1^) for 100 min, followed by strong BL (50 μmol m^−2^ s^−1^) for 80 min, and lastly returned to the dark for 20 min. Leaf transmittance was measured every 2 min, and the transmittance changes between 20 and 30 min after switching to light (the values for 40 to 50 min and 140 to 150 min, respectively) were calculated as leaf transmittance per minute. The transmittance was recorded at 660 nm with a plate reader.

To examine chloroplast movement directly in *Arabidopsis* leaves, the position of chloroplasts was observed after illumination for 2 hours of transgenic *pOpON:AtCDKA;1*(*DN*)-*GFP* plants that had been treated with 20 μM DEX for 24 hours or in WT plants that had been treated with 10 μM AZD5438 for 12 hours. A laser scanning microscope (Zeiss LSM510 META) equipped with a 40×/Korr objective lens was used to excite chlorophyll autofluorescence at 516 nm. Fluorescent signals were captured through narrow-band filters: 640 to 740 nm. Alternatively, images were acquired on an SP8 confocal microscope (Leica Microsystems, Wetzlar, Germany) equipped with an HC PL APO 63×/1.40 oil PH3CS2 objective lens. GFP fluorescence was measured by the time gating method (gating time, 0.5 to 12 ns) to remove chlorophyll autofluorescence, according to a previous study (*63*). Excitation wavelength was 488 nm, and emission was collected between 620 and 685 nm (chloroplast autofluorescence) or between 490 and 550 nm (GFP). For both confocal microscopy imaging analyses, projection images were constructed from *z* stacks using ImageJ.

### Fluorometer fluorescence measurements

Chlorophyll fluorescence was analyzed with a Pulse Amplitude Modulation (PAM-2000) Fluorometer (Heinz Walz GmbH) (*64*). Procedures used for measuring *F*_v_*/F*_m_ were based on standard methodologies, as documented in the PAM-2000 manual.

### Time-gated confocal microscopy and PpCDKA localization

Protonemata were grown in glass-bottom dishes containing BCDATG with 0.5% (w/v) gellan gum in RL (30 μmol m^−2^ s^−1^) for 1 week. Protonemata were then observed on a Leica SP8X confocal microscope. To characterize Dendra2 fluorescence, a time-gated imaging approach was used to exclude chlorophyll autofluorescence (*63*). The objective lens was a 63× oil immersion lens, and the images were 512 × 512 pixels in resolution. GFP was excited with the laser at 488 nm using a white light laser, and the emission spectrum was collected between 495 and 550 nm through an HyD hybrid detector. The gating time was 2.0 to 12.0 ns.

### Phototropic response of *Arabidopsis*

*Arabidopsis* Col-0 seeds harboring pOpON-*AtCDKA;1(DN)-GFP* construct were sown on B5 medium supplemented with 2% (w/v) sucrose and containing 1.5% (w/v) agar, stratified at 4°C for 3 days in the dark, and then exposed to white light for 24 hours. After germination, plates were placed vertically at 22°C in the dark for 2 days, followed by treatment with 30 μM DEX, or the equivalent volume of DMSO for mock treatment, and incubated for 1 day for induction. After confirmation of induction by DEX, as indicated by GFP fluorescence, seedlings were transferred to B5 medium without sucrose and containing 1.5% (w/v) agar in square plates and then illuminated with unilateral BL. Phototropic response was examined by measuring the hypocotyl angle after 2 days of irradiation using ImageJ. To observe the induction by DEX in the hypocotyl, a ZEISS LSM 980 laser scanning microscope equipped with a 40×/water objective lens was used to excite chlorophyll autofluorescence at 639 nm. Autofluorescence was captured between 641 and 694 nm. For the detection of GFP, the excitation wavelength was 488 nm, and the emission was collected between 490 and 596 nm.

## Notes

View/request a protocol for this paper from *Bio-protocol*.
