# Supplementary material for: A PSTAIRE-type cyclin-dependent kinase controls light responses in land plants
Source: Sci Adv. 2022 Jan 28;8(4):eabk2116. doi: 10.1126/sciadv.abk2116 (PMC8797184; doi:10.1126/sciadv.abk2116)
Supplement: Supplementary file 1 — Figs. S1 to S8 Table S1 Legends for movies S1 and S2 Legends for data files S1 to S7 [file sciadv.abk2116_sm.pdf]

Science Advances
AAAS

Supplementary Materials for

A PSTAIRE-type cyclin-dependent kinase controls light responses in land plants

Liang Bao, Natsumi Inoue, Masaki Ishikawa, Eiji Gotoh, Ooi-Kock Teh, Takeshi Higa, Tomoro Morimoto, Eggie Febrianto Ginanjar, Hirofumi Harashima, Natsumi Noda, Masaaki Watahiki, Yuji Hiwatashi, Masami Sekine, Mitsuyasu Hasebe, Masanitsu Wada, Tomomichi Fujita*

*Corresponding author. Email: tfujita@sci.hokudai.ac.jp

Published 28 January 2022, *Sci. Adv.* **8**, eabk2116 (2022)
DOI: 10.1126/sciadv.abk2116

The PDF file includes:

Figs. S1 to S8
Table S1
Legends for movies S1 and S2
Legends for data files S1 to S7

Other Supplementary Material for this manuscript includes the following:

Movies S1 and S2
Data files S1 to S7

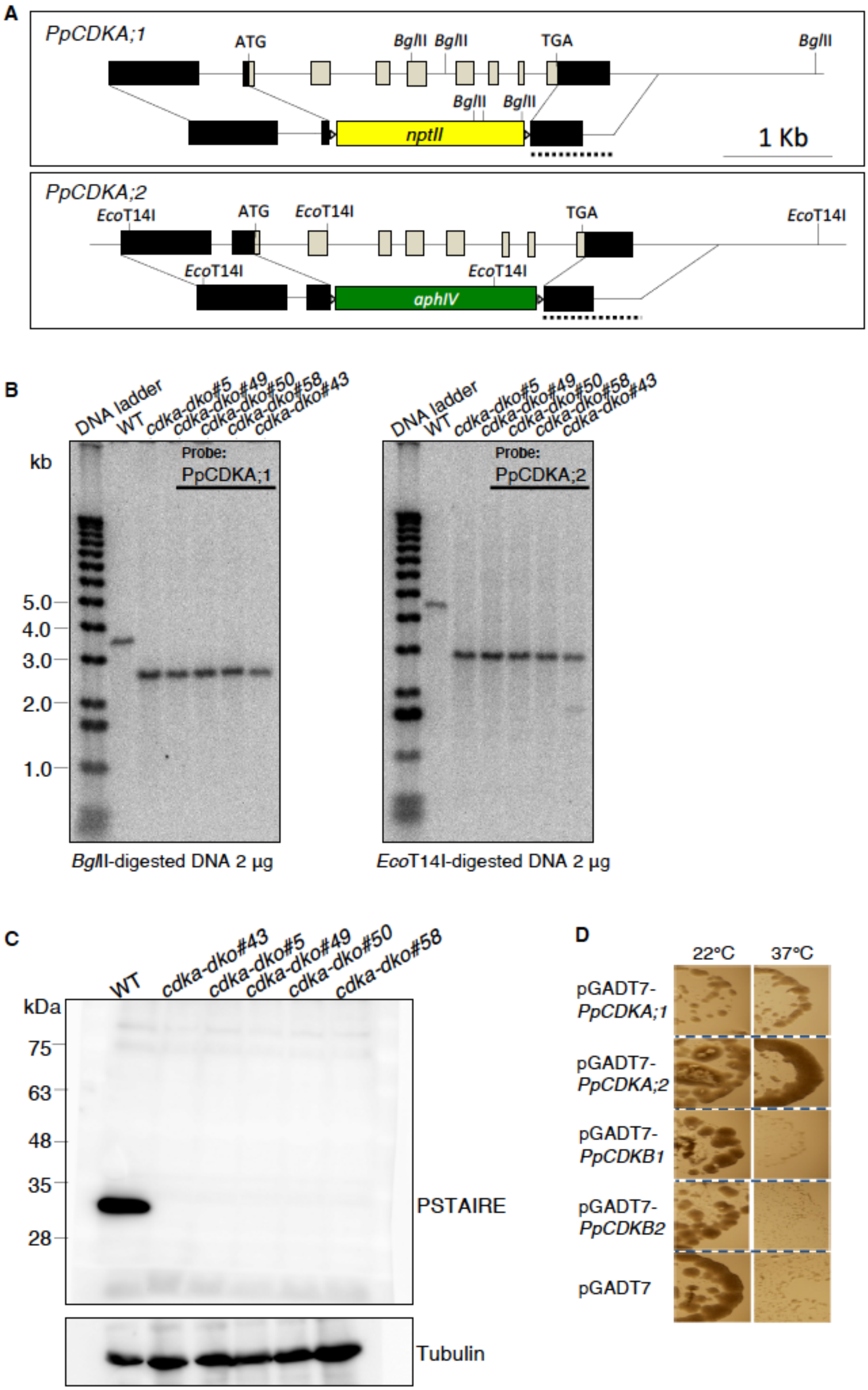
A
PpCDKA;1
ATG
BglII BglII
TGA
BglII
BglII BglII
nptII
1 Kb
PpCDKA;2
EcoT14I
ATG
EcoT14I
TGA
EcoT14I
EcoT14I
EcoT14I
aphIV
B
DNA ladder
WT
cdka-dko#5
cdka-dko#49
cdka-dko#50
cdka-dko#58
cdka-dko#43
kb
Probe:
PpCDKA;1
5.0
4.0
3.0
2.0
1.0
BglII-digested DNA 2 µg
DNA ladder
WT
cdka-dko#5
cdka-dko#49
cdka-dko#50
cdka-dko#58
cdka-dko#43
Probe:
PpCDKA;2
EcoT14I-digested DNA 2 µg
C
WT
cdka-dko#43
cdka-dko#5
cdka-dko#49
cdka-dko#50
cdka-dko#58
kDa
75
63
48
35
28
PSTAIRE
Tubulin
D
22°C
37°C
pGADT7-
PpCDKA;1
pGADT7-
PpCDKA;2
pGADT7-
PpCDKB1
pGADT7-
PpCDKB2
pGADT7

Fig. S1. | Insertion strategy, genetic analyses of the *cdka* double knockout (*cdka-dko*) plants, and complementation of the yeast *cdc28* mutant with *PpCDKA;1* and *PpCDKA;2*.

A, Gene targeting strategy to knock out *PpCDKA;1* and *PpCDKA;2*. The probes (dashed lines) used for Southern blot analyses are indicated. Exons are shown as boxes and introns as lines. Gray and black boxes indicate coding regions and untranslated regions, respectively. The *nptII* cassette confers resistance to G418; the *aphIV* cassette confers resistance to hygromycin. The triangles indicate the *loxP* recombination sites. **B**, Southern blot analysis confirms gene targeting of *PpCDKA;1* (left) and *PpCDKA;2* (right) in the *cdka-dko* lines. **C**, Immunoblot analysis with an anti-PSTAIRE antibody specific for CDKA confirmed the absence of PpCDKA;1 and PpCDKA;2 in *cdka-dko* lines (upper). An anti-tubulin antibody was used as loading control (lower). **D**, Complementation analysis of the *Saccharomyces cerevisiae* temperature-sensitive *cdc28-4* mutant transformed with the empty pGADT7 vector or pGADT7 with *PpCDKA;1*, *PpCDKA;2*, *PpCDKB;1*, or *PpCDKB;2*. The cells were streaked with toothpicks, then grown at the permissive temperature of 22°C (left) or the restrictive temperature of 37°C (right). Note that both *PpCDKA;1* and *PpCDKA;2* complemented *cdc28-4* mutant.

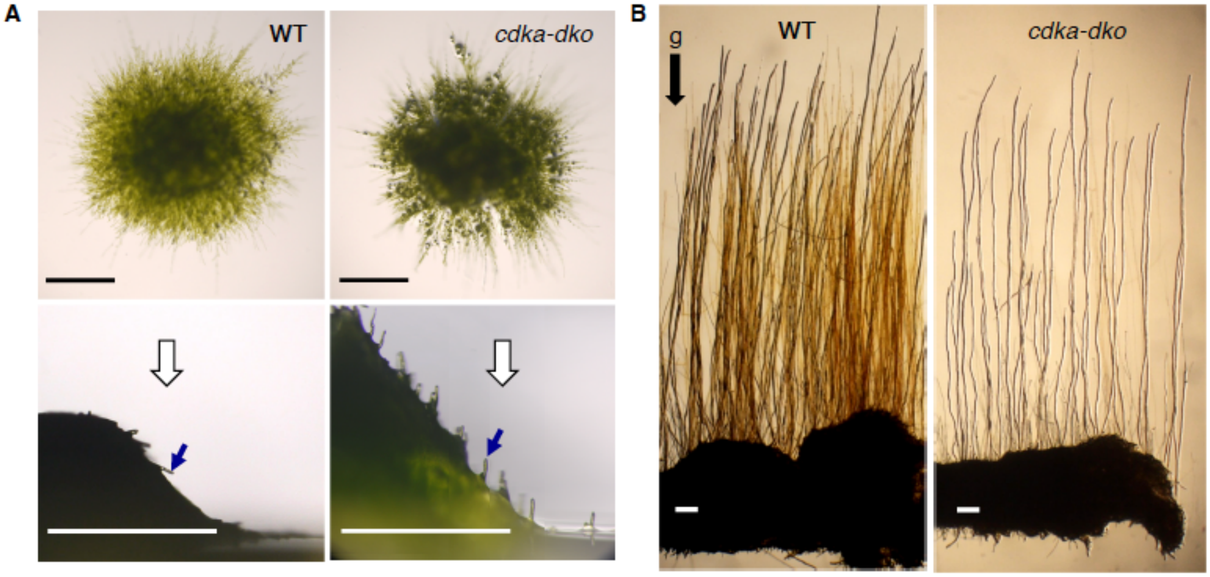

Fig. S2. | PpCDKA controls phototropism but not gravitropism in *P. patens*.

A, Top-down view (upper) and side view (lower) of wild-type (WT, left) and *cdka-dko* (right) plants. The white arrows indicate the direction of white light (~50 µmol m^{-2} s^{-1}). The tips of protonemata (blue arrows) in the WT do not grow toward the light source, whereas those of *cdka-dko* plants do. Scale bars, 1 mm. **B**, Protonemal upward gravitropism in the WT (left) and *cdka-dko* (right) plants is comparable. The plants were grown along the surface of a plate placed vertically in the dark for 34 d. Black arrow indicates the gravity vector. Scale bar, 1 mm.

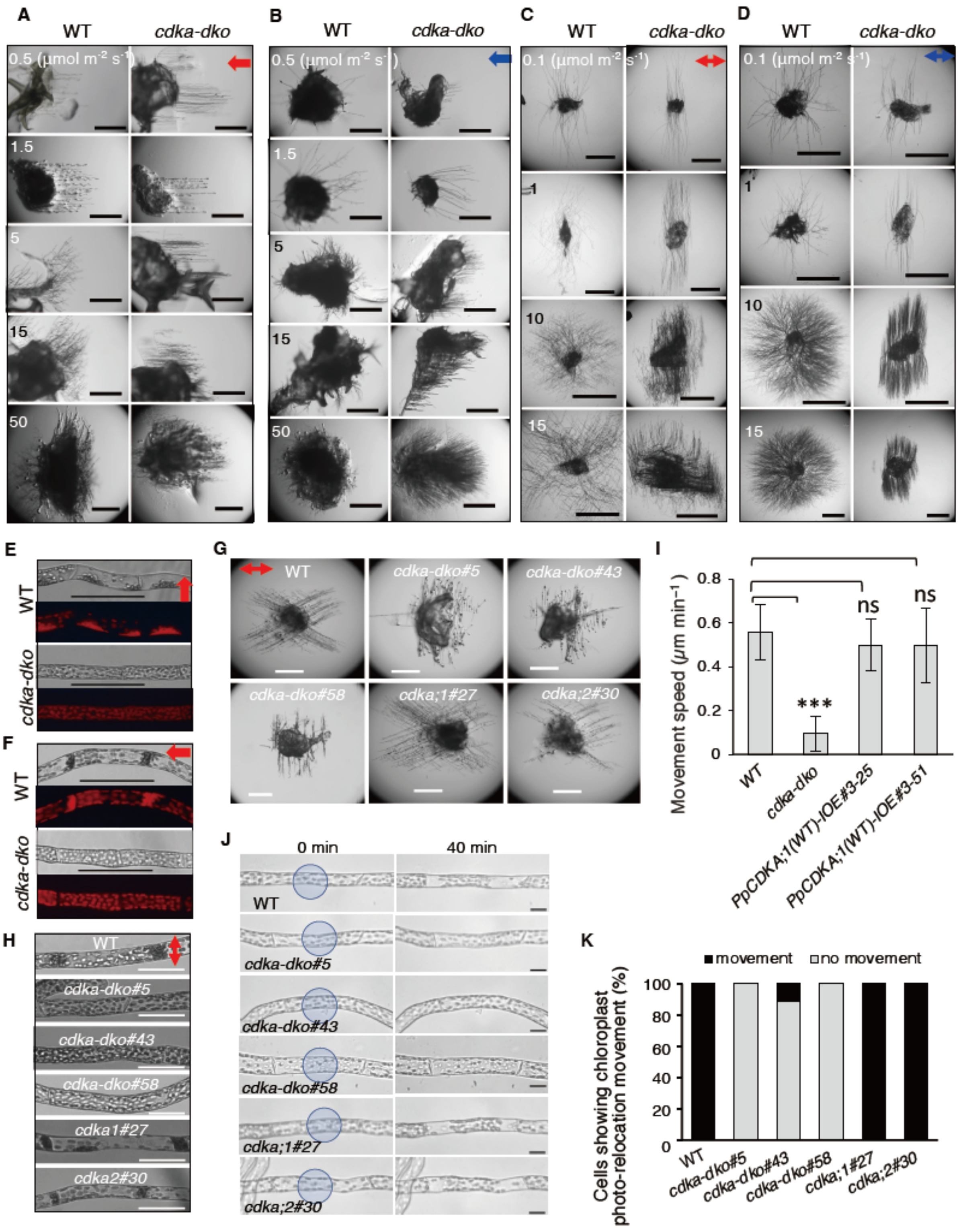

Fig. S3. | PpCDKA;1 and PpCDKA;2 redundantly control phototropism and light-induced chloroplast photo-relocation in *P. patens*.

A, **B**, *PpCDKA* genes regulate phototropism to unilateral red (**A**) and blue (**B**) light. Representative plants are shown after 7 d of light illumination. Scale bars, 2 mm. **C**, **D**, *PpCDKA* genes regulate polarotropism to polarized red (**C**) and blue (**D**) light. Representative plants are shown after 8 d of polarized light illumination. Scale bars, 2 mm. **E**, **F**, Unilateral red light–induced chloroplast movement is controlled by *PpCDKA*. **G**, **H**, PpCDKA;1 and PpCDKA;2 redundantly control light-induced tropism and chloroplast movement. Double-headed arrow indicates the E-vector of polarized red light. Polarotropism phenotypes under polarized red light of 3 µmol m^{-2} s^{-1}. Scale bars, 1 mm (**G**). Chloroplast photo-relocation movement after illumination with polarized red light. Scale bars, 100 µm (**H**). **I**, Induction of a wild-type copy of *PpCDKA;1* (*PpCDKA;1*(*WT*)), without Dendra2 sequence tag, in *cdka-dko* line #5 rescues the movement of blue light–induced chloroplast accumulation to WT levels. Chloroplast accumulation was examined in subapical protonemal cells during illumination with a blue microbeam (0.4 µmol m^{-2} s^{-1}). Data are shown as means ± SD from 15 chloroplasts. Significant differences were determined from a two-tailed Welch's *t*-test. **J**, PpCDKA;1 and PpCDKA;2 redundantly control chloroplast movement, as assayed with a blue microbeam. Scale bars, 20 µm. **K**, Percentages of cells showing chloroplast accumulation in response to the blue microbeam. Data are from three to nine chloroplasts.

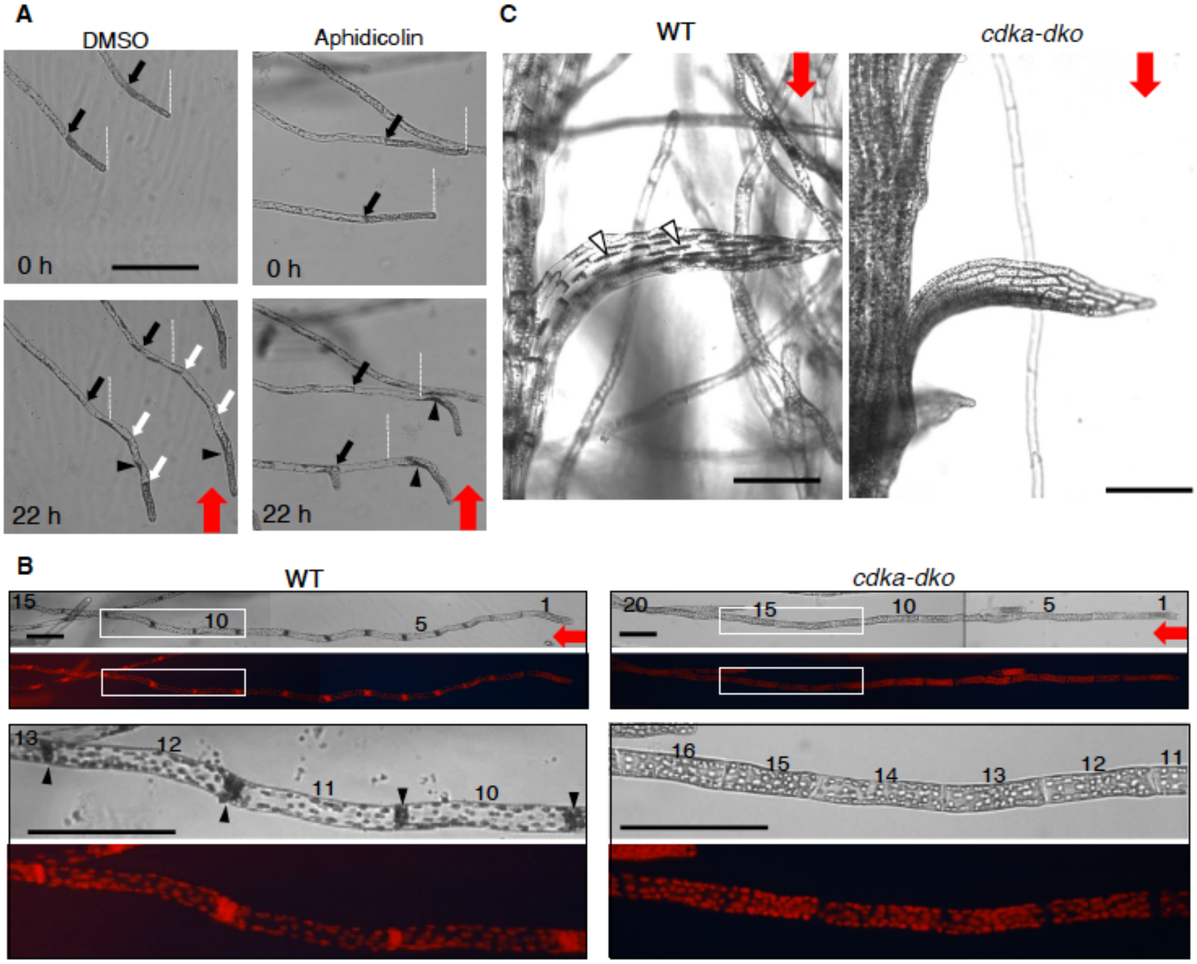

Fig. S4. | PpCDKAs control chloroplast photo-relocation movement independently of the cell cycle in *P. patens*.

A, Phototropism and chloroplast movement in aphidicolin-treated wild-type (WT) protonema cells. WT plants were treated with DMSO only or with 10 μg/mL aphidicolin for 22 h while being illuminated with unilateral red light. Note that cell division is inhibited by aphidicolin treatment. Dashed lines indicate the tip positions of apical cells at time 0 h; black arrows indicate the septum of apical-subapical cells at time 0 h; white arrows indicate the septum produced by newly divided cells; black arrowheads indicate chloroplast accumulation. Scale bar, 100 μm. **B**, Requirement of

PpCDKAs for chloroplast movement in dividing cells (ca. from the 1st to the 8th position) and in non-dividing cells (ca. from the 9th and more basal position) in both the apical and basal protonemal tissues, respectively. Chloroplast movement was examined with constant unilateral red light (red arrows) at a fluence rate of 0.5 µmol m^{-2} s^{-1}. The numbers indicate cell positions along protonemal filaments. White rectangles indicate magnified basal regions (bottom two panels). Red, chlorophyll autofluorescence. Black arrowheads indicate the regions of chloroplast accumulation. Scale bars, 100 µm. **C**, Requirement of PpCDKAs for chloroplast movement in non-dividing cells in fully differentiated, gametophytic leaves. Chloroplast distribution was examined under unilateral red light (red arrows). Note that chloroplasts are evenly distributed in *cdka-dko* cells. White arrowheads indicate chloroplast accumulation. Scale bars, 1 mm.

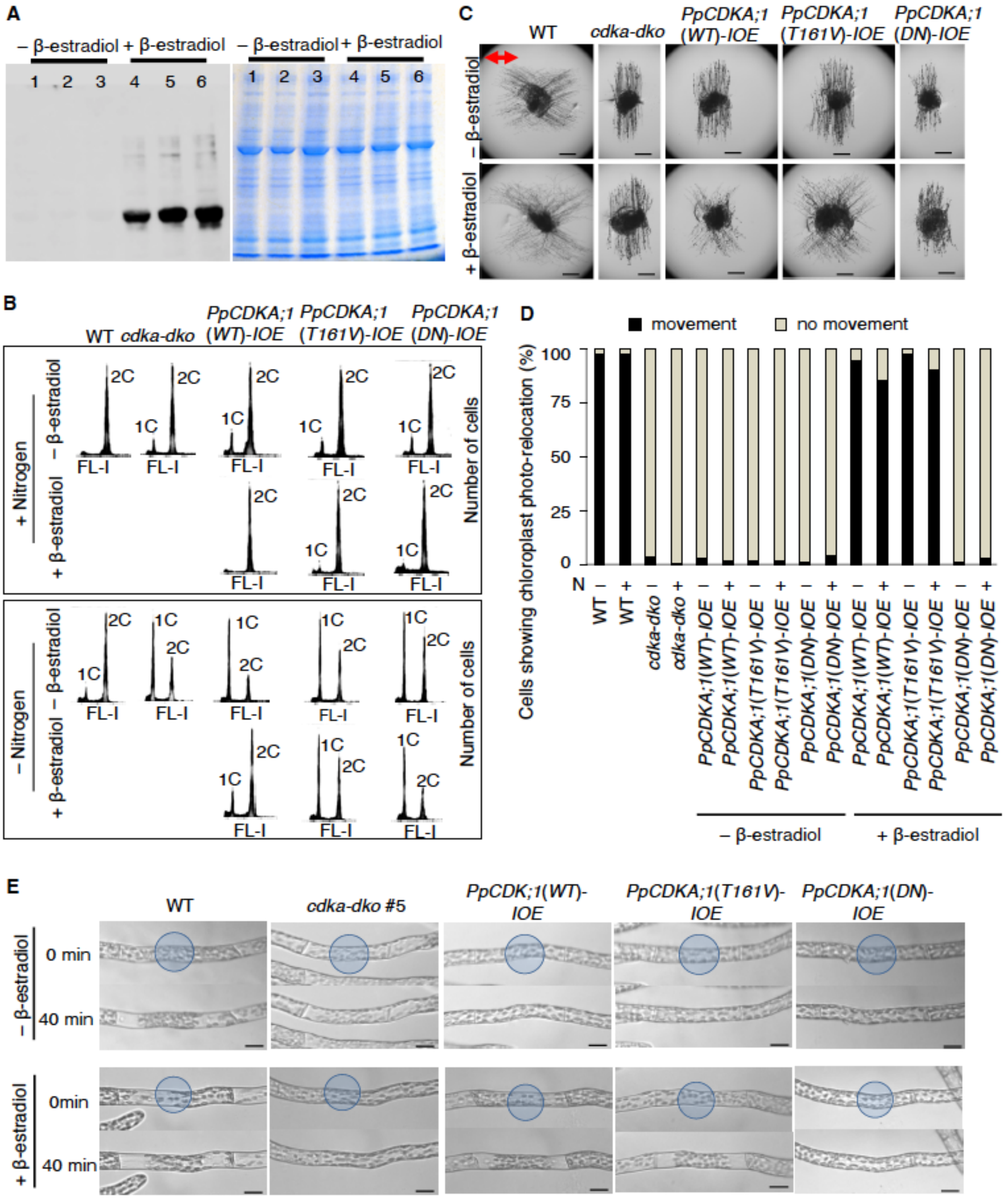

Fig. S5. | PpCDKA kinase activity is required for cell cycle progression, phototropism, and light-induced chloroplast photo-relocation movement.

A, Immunoblot analysis of CDKA protein levels in *PpCDKA;1*(*WT*)-*IOE* (lanes 1, 4), *PpCDKA;1*(*T161V*)-*IOE* (lanes 2, 5), and *PpCDKA;1*(*DN*)-*IOE* (lanes 3, 6) plants with and without β-estradiol induction using anti-Dendra2 antibody (left). Plants were grown in constant white light (50 µmol m^{-2} s^{-1}) for 5 d, followed by 1 d in polarized red light (3 µmol m^{-2} s^{-1}). Coomassie Brilliant Blue (CBB) staining was used as loading control (right). **B**, Ploidy profiles of *cdka-dko* mutants transformed with *PpCDKA;1* constructs. Plants were grown in medium containing nitrogen (+ Nitrogen, upper) or lacking nitrogen (–Nitrogen, lower) in constant white light (50 µmol m^{-2} s^{-1}) for 5 d, followed by 1 d in polarized red light (3 µmol m^{-2} s^{-1}). Note that only *PpCDKA;1*(*WT*) rescues the cell cycle defect of *cdka-dko* plants upon induction with β-estradiol. **C**, **D,** Rescue of the polarotropism (**C**) and chloroplast movement (**D**) defect of *cdka-dko* plants by *PpCDKA;1*(*WT*) and *PpCDKA;1*(*T161V*), but not by *PpCDKA;1*(*DN*). Scale bars, 1 mm (**C**). Percentage of cells (*n* = 109–196 each) showing chloroplast accumulation (**D**). **E**, Representative images of chloroplast movement induced by a blue microbeam for 40 min in plants grown on nitrogen-deficient medium. Scale bars, 20 µm.

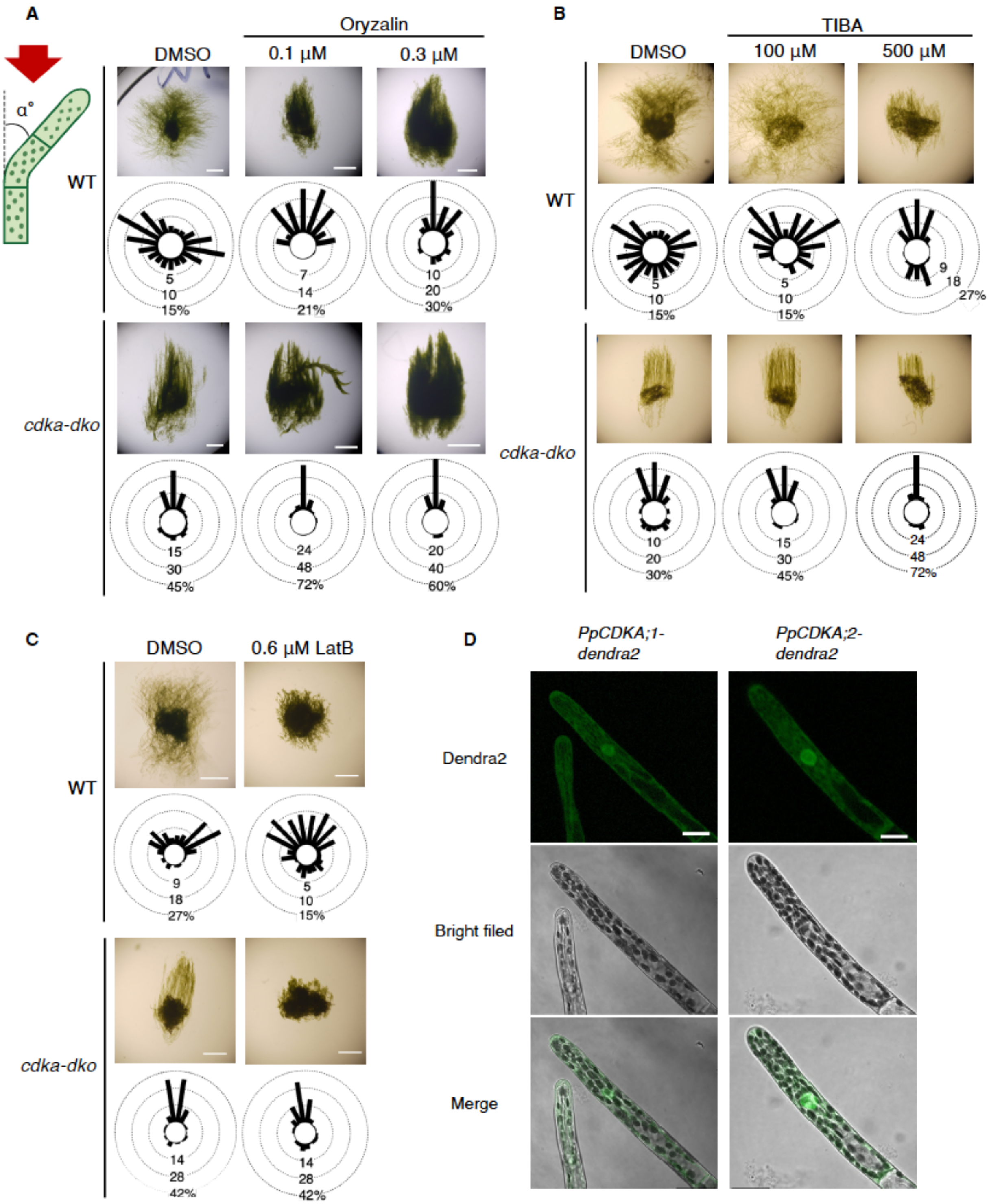

Fig. S6. | The cytoskeleton components, microtubules, and microfilaments are involved in PpCDKA-mediated phototropism.

A–C, Phototropism of protonemata that were illuminated with unidirectional red light (red arrow). Protonemata were cultured with oryzalin, an inhibitor of microtubule polymerization (**A**); treated with TIBA, which enhances actin bundling (**B**); or treated with latrunculin B, an actin polymerization inhibitor (**C**). The angles indicated are between the apical direction of protonemata and the direction of incident light (α) and are shown as a circular histogram. Protonemata of *cdka-dko* plants adopted the same direction of growth in all treatments. Scale bars, 1 mm. **D,** Subcellular localization of PpCDKA;1-Dendra2 and PpCDKA;2-Dendra2 using time-gated confocal microscopy. The Dendra2 signal was detected in the nucleus and in the cytosol of protonemata.

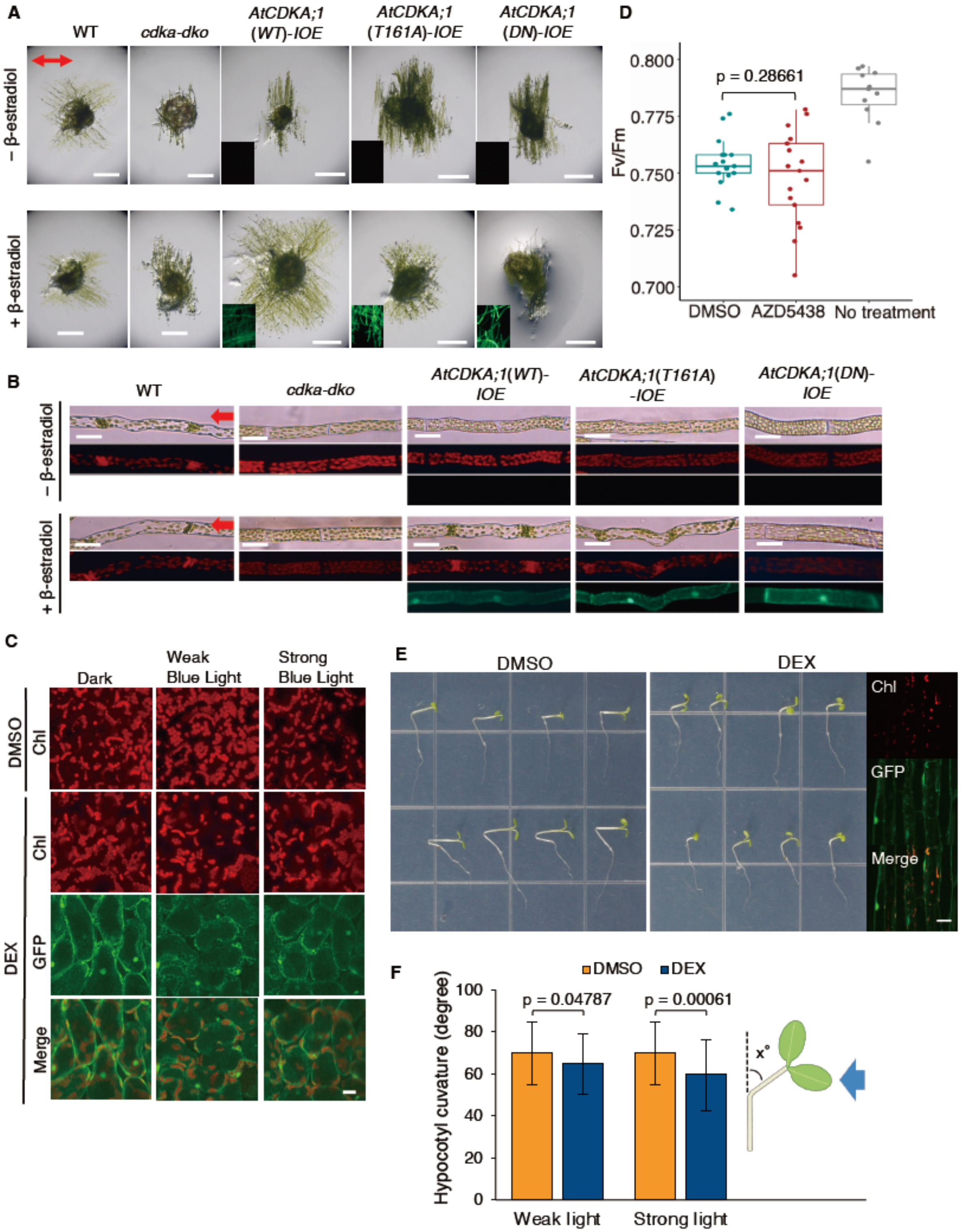

Fig. S7. | AtCDKA;1 controls light-induced tropism and chloroplast accumulation response in *P. patens* and Arabidopsis.

A, B, *AtCDKA;1*(*WT*) and *AtCDKA;1*(*T161A*), but not *AtCDKA;1*(*DN*), rescue the phototropism (**A**) and chloroplast accumulation (**B**) defect of *cdka-dko* plants. Plants were grown in medium with 0.02% (v/v) DMSO (upper) or 1 μM β-estradiol (lower). Insets show GFP fluorescence for induction of protein accumulation (**A**). Scale bars, 1 mm (**A**) or 0.1 mm (**B**). **C**, Chloroplast distribution in Arabidopsis plants grown in different conditions (dark, weak blue light [3 μmol m^{-2} s^{-1}] or strong blue light [50 μmol m^{-2} s^{-1}]). Leaves of transgenic *AtCDKA;1*(*DN*)-*IOE* plants were treated with 0.1% (v/v) DMSO or 20 μM dexamethasone (DEX). Chl, chlorophyll; GFP, AtCDKA;1(DN)-GFP. Scale bar, 20 μm. **D**, Boxplot of maximum photochemical efficiency of photosystem II (PSII; F_v/F_m) measured in leaves treated with DMSO or 10 μM AZD5438, or receiving no treatment (*n* = at least 11 leaves). *P*-value is from a two-tailed Welch's t-test. **E**, Phototropic responses of the Arabidopsis hypocotyl are reduced in *AtCDKA;1*(*DN*)-*IOE* transgenic seedlings. Seedlings were grown on medium containing 0.1% DMSO or 30 μM DEX and exposed to 50 μmol m^{-2} s^{-1} of unilateral blue light (blue arrow in **F**) for 2 d. Chl, chlorophyll; GFP, AtCDKA;1(DN)-GFP. **F**, Hypocotyl curvature, shown as mean ± SD (*n* = 15–24 seedlings). *P*-values are from a two-tailed Welch's *t*-test.

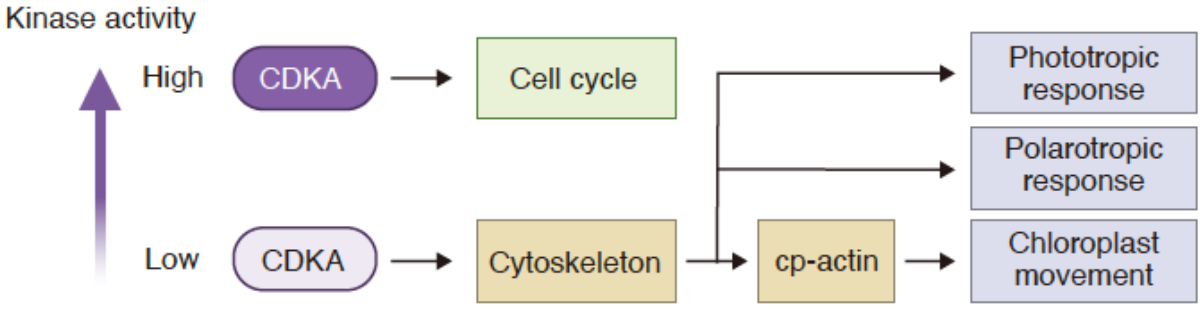

Fig. S8. | Model of CDKA function in light-induced tropism and chloroplast photo-relocation movement in plants.

CDKAs not only control cell cycle progression but also play key roles in light responses, i.e., phototropism, polarotropism, and chloroplast photo-relocation movements. CDKA-mediated light responses are independent of cell cycle regulation, and low levels of kinase activity are sufficient to affect light responses. CDKA-mediated light responses likely act by controlling the actin- and microtubule-based cytoskeletons.

Table S1. Primers used in this study.

Primer name	Sequence (5′ → 3′)	Comments
PpCDKA14-start	CACCATGGATCAGTATGAGAAAGTGGAGA	pENTR-*PpCDKA;1*(*WT,T161V, DN*), *PpCDKA;1*(*T161V*) overlapping PCR, *PpCDKA;1*(*WT,T161V, DN*)-*dendra2* overlapping PCR
PpCDKA14-end	GGGTACAAGACCGATATCCTTG	pENTR-*PpCDKA;1*(*WT,T161V, DN*)
PpCDKA8-start	CACCATGGAACAGTATGAAAAAGTGGAGA	pENTR-*PpCDKA;2*
PpCDKA8-end	CGGCACCAGACCGACA	pENTR-*PpCDKA;2*
PpCDKB(pdp71223)-F	CACCATGTCTGTTTCTGGTAT	pENTR-*PpCDKB;1*
Pp CDKB(pdp71223)-R	TTAGGTTGCTGTCTTATCAACAT	pENTR-*PpCDKB;1*
PpCDKB(pdp71841)-F	CACCATGTCGGGGATGGACAA	pENTR-*PpCDKB;2*
PpCDKB(pdp71841)-R	TTAAGCAGAGGTTTTGTCGAA	pENTR-*PpCDKB;2*
Ppcdka14-5f	CCAGCCTCGTATCTGACTGCCTTGTC	*PpCDKA;1-ko*
Ppcdka14-5r	GATGGAGTACCAGCTCCGCACAAAAC	*PpCDKA;1-ko*
Ppcdka14-3f	GATTGCCTCCGTCGCCTTTCCTACT	*PpCDKA;1-ko*, probe for DNA gel-blot analysis
Ppcdka14-3r	GGTCGTTTTCACCTTCGTCCGTTTG	*PpCDKA;1-ko*
Pp cdka8-5f	CGTGGAATTGCGGCATGTAAGTTTG	*PpCDKA;2-ko*
Ppcdka8-5r	TCCAAAAGCCGAATCACGGAAAGTC	*PpCDKA;2-ko*
Ppcdka8-3f	CCCCCTGACAAAGGCTGAAGAACATT	*PpCDKA;2-ko*, probe for DNA gel-blot analysis
Ppcdka8-3r	AGCGCCGGACCCAACTCTGTCTATTT	*PpCDKA;2-ko*
PpCDKA-T161V-5R	CAATGTTACCACCTCATGAACGAATGTCCTGACAGGAATAC	*PpCDKA;1*(*T161V*) overlapping PCR
PpCDKA-T161V-3F	GTATTCCTGTCAGGACATTCGTTCATGAGGTGGTAACATTG	*PpCDKA;1*(*T161V*) overlapping PCR
PpCDKA14-end2	TCAGGGTACAAGACCGATATCC	*PpCDKA;1*(*T161V*) overlapping PCR
PpCDKA-Dendra2-R	CAGGTTAATTCCCGGGGTGTTGGGTACAAGACCGATATCCTTG	*PpCDKA;1*(*WT,T161V, DN*)-*dendra2* overlapping PCR
PpCDKA-Dendra2-F	CAAGGATATCGGTCTTGTACCCAACACCCCGGGAATTAACCTG	*PpCDKA;1*(*WT,T161V, DN*)-*dendra2* overlapping PCR
Dendra_C_r1	TTAAGCTTGAGCTCGAGTCTTGTAC	*PpCDKA;1*(*WT,T161V, DN*)-*dendra2* overlapping PCR
AtCDKA;1-F	CACCATGGATCAGTACGAGAAAGTTG	*AtCDKA;1*(*WT,T161A,DN*)-GFP

Primer name	Sequence (5′ → 3′)	Comments
sGFP-reverse	TTACTTGTACAGCTCGTCCATGCC	*AtCDKA;1*(*WT,T161A,DN*)-GFP
5’F infusion primer (Xho1)	ACCGGGCCCCCCCTCGAGTATGGATTTACTGAAACTACTTTA	*PpCDKA;1* 5’-flanking in GFP-mTalin
5’R infusion primer (Sal1)	CTTATCGATACCGTCGACCAACATCACTCTCCATGTACATGA	*PpCDKA;1* 5’-flanking in GFP-mTalin
3’F infusion primer (Xba1)	CGCATGCCCGGGTCTAGATACCAGACAAAACGAACAAACGGA	*PpCDKA;1* 3’-flanking in GFP-mTalin
3’R infusion primer (Not1)	CACCGCGGTGGCGGCCGCATGCTGGCATTCGTGCTGGTGCAA	*PpCDKA;1* 3’-flanking in GFP-mTalin
5’F infusion primer (Xho1)	ACCGGGCCCCCCCTCGAGAAGAACTTATGGGCCTCAGAAAGA	*PpCDKA;2* 5’-flanking in GFP-mTalin
5’R infusion primer (Hind3)	GAATTCGATATCAAGCTTAAGCAGCAACCACACCTTTGAGCA	*PpCDKA;2* 5’-flanking in GFP-mTalin
3’F infusion primer (Xba1)	GGATCCACTAGTTCTAGAACTACTTCAAACTCACTTGGTGCT	*PpCDKA;2* 3’-flanking in GFP-mTalin
3’R infusion primer (Not1)	CACCGCGGTGGCGGCCGCCAGCTTCCAATCAAATGATGATAT	*PpCDKA;2* 3’-flanking in GFP-mTalin
pTN182-Hyg-F	ATCGATAAGCTTGATATCAAGTGGATTGATGTGACATCTCCA	Modify pTN182 by switching *nptII* to *aphIV*
pTN182-Hyg-R2	ATCTTCTTACCTTGCACAGGTGCCTTAGCATCTTTTCCCTAACG	Modify pTN182 by switching *nptII* to *aphIV*
pCDFDuet-MBP-infusion-F	CAGGATCCGAATTCGAGCTCATGAAAATCGAAGAAGGTAA	pCDFDuet-MBP-PpCDKA;1-GST-Cak1
pCDFDuet-CDKA (stop)-R	TTATGCGGCCGCAAGCTTTCAGGGTACAAGACCGATATC	pCDFDuet-MBP-PpCDKA;1-GST-Cak1
CDKA-infusion-F	ATTTCAGAATTCGGATCCATGGATCAGTATGAGAAAGTGGA	pMAL-c2X-MBP-PpCYCD;2
CDKA-infusion-R	ACGGCCAGTGCCAAGCTTTCAGGGTACAAGACCGATATC	pMAL-c2X-MBP-PpCYCD;2

Movie S1. | Chloroplast accumulation response in wild-type *P. patens*.

Representative chloroplast accumulation response in wild-type *P. patens*. Representative time-lapse movie showing the movement of chloroplasts towards the illuminated area in the WT. Subapical cells were used for illumination for 30 min with a blue light microbeam (0.4 µmol m^{-2} s^{-1}); cells were imaged every 1 min during illumination.

Movie S2. | Chloroplast accumulation response in *cdka-dko* plants.

Representative time-lapse movie showing the movement of chloroplasts towards the illuminated area in *cdka-dko* plants. Subapical cells were used for illumination for 30 min with a blue light microbeam (0.4 µmol m^{-2} s^{-1}); cells were imaged every 1 min during illumination.

Data file S1. | Data set used in Fig. 1E to 1I and Fig. 2D (separate excel file).

Data file S2. | Data set used in Fig. 3D and 3F (separate excel file).

Data file S3. | Data set used in Fig. 4C to 4F (separate excel file).

Data file S4. | Data set used in Fig. S3I and S3K (separate excel file).

Data file S5. | Data set used in Fig. S5D (separate excel file).

Data file S6. | Data set used in Fig. S6A to S6C (separate excel file).

Data file S7. | Data set used in Fig. S7D and S7F (separate excel file).
